# Small population bias and sampling effects in stochastic mortality modelling

**DOI:** 10.1007/s13385-016-0143-x

**Published:** 2017-01-23

**Authors:** Liang Chen, Andrew J. G. Cairns, Torsten Kleinow

**Affiliations:** Actuarial Research Centre, Department of Actuarial Mathematics and Statistics and the Maxwell Institute for Mathematical Sciences, School of Mathematical and Computer Sciences, Heriot-Watt University, EH14 4AS Edinburgh, UK

**Keywords:** Small population, Age effect, Period effect, Cohort effect, Bootstrap, Parameter uncertainty, Systematic parameter difference, Likelihood ratio test, Power of test

## Abstract

We propose the use of parametric bootstrap methods to investigate the finite sample distribution of the maximum likelihood estimator for the parameter vector of a stochastic mortality model. Particular emphasis is placed on the effect that the size of the underlying population has on the distribution of the MLE in finite samples, and on the dependency structure of the resulting estimator: that is, the dependencies between estimators for the age, period and cohort effects in our model. In addition, we study the distribution of a likelihood ratio test statistic where we test a null hypothesis about the true parameters in our model. Finally, we apply the LRT to the cohort effects estimated from observed mortality rates for females in England and Wales and males in Scotland.

## Introduction

Stochastic mortality models are widely used as risk management tools in the insurance and pensions industry with the main application being the generation of plausible scenarios for future mortality rates. Many stochastic mortality models have been introduced in the last few decades. When new models have been developed the objective was mostly to improve the goodness of fit of the model to mortality data observed in relatively large populations: the Lee-Carter model and its refinements (e.g. [3, 23, 31]) have been developed to provide a good fit to the mortality rates observed in the United States, England and Wales and the population of UK male assured lives; while the Cairns-Blake-Dowd ([6]) model (CBD) was introduced for modelling the England and Wales males population at higher ages.

In contrast, actuaries will often face the problem of modelling the mortality experience of much smaller populations, for example, the members of a pension scheme. Empirical research has found that mortality rates of smaller populations exhibit significantly more variability compared to the observed rates in larger populations. Furthermore, models that fit large countries well, might not be appropriate for smaller populations, for example, [3] showed that the Lee-Carter model provides a rather poor fit to the mortality experience of smaller populations. A related issue is that empirical data from smaller populations might only be available for a relatively short period, which makes mortality projections rather uncertain. As a result, a number of recent papers have aimed to develop models specifically for smaller populations: for example, the Saint Model of [18].

A common assumption for many of the proposed models is that the observed numbers of deaths are realisations of random variables with a Poisson distribution given the underlying mortality rates. The estimation of parameters of any such model is therefore based on samples from a Poisson distribution, and, as always in statistics, parameter uncertainty is related to the sample size. Furthermore, many results about the distribution of estimators and corresponding confidence intervals rely on the Maximum Likelihood theorem and large sample sizes.

The increased uncertainty about estimated parameters for small populations results in high levels of uncertainty about projected mortality rates. As a consequence future realised mortality rates will not only diverge from projected rates due to future sampling variation caused by the Poisson distribution, but might also diverge from projections since the projections themselves are uncertain.

In the actuarial literature, simulation techniques have been proposed for dealing with uncertain parameters and projected mortality rates. For example, [24] investigated mortality uncertainty by applying a block bootstrap method on the Lee-Carter model, and [4] proposed Poisson bootstrap methods for mortality forecasting. [6] studied the parameter uncertainty of the two factor CBD model by adopting a Bayesian approach. Czado et al. and Pedroza [12, 29] carried out the first Bayesian analysis using Markov Chain Monte Carlo (MCMC) of the Lee-Carter model, with further work by [21, 22]. Reichmuth and Sarferaz [30] applied MCMC to a version of the [31] model. Cairns et al. (2011) applied MCMC to a two-population Age-Period-Cohort model by combining the Poisson likelihood for the deaths counts with time series likelihood functions for the latent random period and cohort effects.

However, to the best of our knowledge, bootstrap methods have not been applied in a systematic way to investigate the impact of the size of a population on parameter and projection uncertainty. This is the focus of our research in this paper. We firstly apply Poisson parametric bootstrap methods to investigate how the variation of parameter estimates and projections is affected by the size of a population. The specific mortality model that we consider is a second generation CBD model with added cohort effect: see Sect. 2 for details. We vary the size of the population by assigning weights to a chosen benchmark population, e.g. England and Wales males. In simulation studies we find that the size of the population has a significant effect on the variation of parameter estimates and projections.

Although we apply a weight to the benchmark population (i.e. scale it down), we ensure that the mortality rates of the constructed small populations are equal to the fitted mortality rates of the benchmark population. In such a situation, uncertainty in projected mortality rates will be reduced if information from the benchmark population parameter estimates can be used for fitting smaller populations. This raises the question of how we can test for systematic differences between the parameters driving mortality rates in a small population and a given null hypothesis about those parameters, where the null hypothesis might have been obtained from a model fitted to a much larger population. If no significant differences can be found then it seems reasonable to use elements of the large population model fit to assist in generation of scenarios for the small population. We therefore investigate the properties of a likelihood ratio (LR) test for all or some of the estimated parameters, and, in particular, consider the distribution of the test statistic based on the bootstrap simulations. This allows us to investigate the power of the LR test and the effect of varying population sizes on the rejection rates. We find that the population size has a strong effect on the probability of a type II error. This is particularly relevant for pension schemes since the acceptance of an incorrect null hypothesis might lead to inaccurate mortality assumptions. To investigate the financial consequences of the resulting misspecified model, we consider annuity prices based on different assumptions about the underlying parameters of our model.

We apply the LR test in an empirical study. The null hypothesis for that study is the estimated cohort effect for males in England and Wales. With this null hypothesis we then carry out hypothesis tests using, first, mortality data for females in England and Wales and, second, males in Scotland to check if their cohort effects are significantly different from the estimated cohort effect for males in England and Wales. We find for both populations that the estimated cohort effect is significantly different from that in the null hypothesis.

The remainder of the paper is organised as follows. Section 2 introduces the model, assumptions and the notations we apply. Section 3 discusses the process of simulation and investigates the distribution of the maximum likelihood estimates, the correlation between the estimates and how these will be affected by changing the population size. In Sect. 4, we investigate the effect of the population size on forecasting by projecting the parameters as well as the mortality rates. Section 5 introduces a likelihood ratio test for testing systematic deviations of the true parameters from a given null hypothesis. The power of the likelihood ratio test is also analysed and we then investigate how significant the impact of shifting and scaling parameters is on the fitted mortality rates and corresponding annuity prices in Sect. 6. Finally, Sects. 7 and 8 include the LRT for testing a null hypothesis about the cohort effect only, and an empirical example for this test is provided. Section 9 provides our final conclusions.

## The model

We denote by *D*(*t*, *x*) the number of deaths during calendar year $t=t_1,\ldots ,t_{n_y}$ at age $x=x_1,\ldots ,x_{n_a}$ and by *E*(*t*, *x*) the corresponding central exposure to risk.

We will fit the following Poisson model to the observed death data, see [8]:

1 $$\begin{aligned} D(t,x) | \theta\sim & {} \text {Pois}(m(\theta , t,x) E(t,x)) \end{aligned}$$

2 $$\begin{aligned} m(\theta , t,x)= & {} - \log (1-q(\theta , t,x)) \end{aligned}$$

3 $$\begin{aligned} \text {logit}\;q(\theta ,t,x)= \kappa _t^{(1)} + \kappa _t^{(2)}(x-\bar{x}) + \kappa _t^{(3)}((x-\bar{x})^2-{\hat{\sigma }}_x^2) +\gamma _{t-x}^{(4)} \end{aligned}$$

where the parameter vector $\theta$ is given by

$$\begin{aligned} \theta =(\kappa _t^{(1)},\kappa _t^{(2)},\kappa _t^{(3)},\gamma _c^{(4)}) \end{aligned}$$

with the following interpretations:

- $\kappa _t^{(i)}$ is a period effect in year $t=t_1,\ldots ,t_{n_y}$ for each $i = 1,2,3$,
- $$\kappa =\{\kappa ^{(1)},\kappa ^{(2)},\kappa ^{(3)}\}$$, where $$\kappa ^{(i)}=\{\kappa _t^{(i)}\}_{t=t_1,\ldots t_{n_y}}$$ for $$i=1, 2, 3$$,
- $\gamma _c^{(4)}$ is the cohort effect for the cohort born in year $c=t-x$,
- $$\gamma ^{(4)}=\{\gamma _c^{(4)}\}_{c=t_1-x_{n_a},\ldots ,t_{n_y}-x_1}$$
- $\bar{x}$ is the mean of the age range we use for our analysis, and
- ${\hat{\sigma }}_x^2$ is the mean of $(x-\bar{x})^2$.

The reason for including the cohort effect is that it is a well established feature in some populations such as England and Wales [8]. We do not claim that this model is necessarily the best model for the datasets to be considered. However we select the model based on a particular set of model selection criterion studied in Ref. [8]. The choice of “M7” here reflects the work of Ref. [8] namely that we want to use a model that fits the males from England and Wales well.

It is well known that the parameters in model (3) are not identifiable without imposing constraints on their values. Nielsen and Nielsen [27] discussed the impact of identifiability problems within stochastic mortality models on parameter estimation, hypothesis testing and forecasting. Currie [11] discussed modelling with M7 by writing the model as a generalized linear model with a non-full rank design matrix. We follow Ref. [8] and apply the following constraints on $\theta$:

4 $$\begin{aligned} \sum _{c\in C}^{} \gamma _c^{(4)} = 0, \qquad \sum _{c\in C}^{} c\gamma _c^{(4)} = 0, \qquad \sum _{c\in C}^{} c^2\gamma _c^{(4)} = 0 \end{aligned}$$

where $C=t_1 - x_{n_a} , \ldots , t_{n_y}-x_1$ is the set of all years of birth in a given dataset. In this study, identifiability constraints are defined as part of our model system to ensure all parameters are identifiable and provide a coherent framework for the consideration of confidence intervals and for hypothesis testing. One can freely adopt any reasonable set of constraints to the model and the study would be focusing on the results given the selected constraints.

To estimate the parameters in (3) we apply maximum likelihood estimation. The log-likelihood function in our model is

5 $$\begin{aligned} l(\theta ;D,E)=\sum _{t,x}D(t,x)\text {log}[E(t,x)m(\theta ,t,x)]-E(t,x)m(\theta ,t,x)-\text {log}[D(t,x)!] \end{aligned}$$

where $m(\theta ,t,x)$ is given by (2) and (3). It is worth noticing that both the fitted mortality rates and the log-likelihood function $l(\theta ;D,E)$ are invariant to the choice of the identifiability constraints.

As mentioned earlier, in this paper we are concerned with the consequences of small exposures, or population sizes, on the distribution of the maximum likelihood estimator (MLE) ${\hat{\theta }}$ of $\theta$. To study the distribution of the MLE ${\hat{\theta }}$ we will simulate death data *D*(*t*, *x*) from the model in (1–3) using a given parameter vector $\theta _0$ and different exposure sizes.

To ensure that our results are relevant for typical values of $\theta$ we first fit our model to death and exposure data observed in England and Wales during the years 1961 to 2011 for males aged 50 to 89. Note that we do not claim that this is the only choice of dataset. Any large population plus any model that is known to fit it well can be used for this study. The reason for the choice of dataset is that we have familiarity with the England and Wales data and the selected model fits the similar dataset well in Ref. [8]. We then fix $\theta _0$ to be equal to the estimated parameter vector ${\hat{\theta }}^{\text{ EW }}$ for this data. Note that this is only an example for the true parameter vector $\theta _0$ and our analysis can be applied to other choices of $\theta _0$. Mortality data for England and Wales are obtained from the Human Mortality Database.^1^ Note that we do not exclude short cohorts from the estimation since we are interested in how the MLE fits the short cohorts and the impact of small population sizes on the estimates.

The different exposure sizes used to simulate data in the remainder of this paper will be relative to the exposure $E_0(t,x)$ for a benchmark population. For reasons of practical relevance and consistency with our choice of $\theta _0$ the benchmark population is the male population in England and Wales unless stated otherwise.

## Distribution of MLE in finite samples

For any given parameter vector $\theta _0$ and benchmark exposure $E_0(t,x)$ we define the small-sample exposure as

$$\begin{aligned} E^w(t,x)= w E_0(t,x) \end{aligned}$$

for a constant $w \le 1$. Table 1 shows the exposures for males in England and Wales in our dataset in year 2011 with selected ages (50, 60, 70, 80, 89). The total exposure for males in England and Wales in 2011 across all ages from 50 to 89 is 9, 049, 613. The weights we consider in this paper are 1, 0.1, 0.01 and 0.001. The smallest population will therefore have an exposure of 43 at age 89 and 382 at age 50.

**Table 1:** The exposure of males in England and Wales (EW) in the dataset in year 2011 with selected ages (50, 60, 70, 80, 89)

Age *x*		50	60	70	80	89
Exposure	EW	381, 797	307, 825	213, 455	134, 966	42, 640

We then simulate *N* scenarios for the death counts $D^w(t,x)$ using the model in (1–3) with $\theta = \theta _0$. Through our simulation we obtain *N* independent scenarios $D_j^w(t,x)$ for the death counts with

6 $$\begin{aligned} D_j^w(t,x) \sim \text {Pois}\big (m(\theta _0, t, x) w E_0(t,x) \big ) \quad \text{ for } \text{ all } j = 1, \ldots , N. \end{aligned}$$

A more general approach would be to consider a weights matrix $W=\{w_{t,x}\}$ allowing for weights to depend on age and calendar year. This would be particularly relevant when our proposed methodology is applied to investigate the mortality of members of a pension scheme with a very different age structure than the age structure of the overall population in England and Wales. However, for clarity of presentation, we only consider a constant weight applied to all ages and calendar years.

### MLE

To obtain MLEs for ${\hat{\theta }}^w_j$ for each simulated scenario *j* and each *w* we maximise the log-likelihood function $l(\theta ;D_j^w,E^w)$ as given in (5) subject to the constraints in (4), that is,

7 $$\begin{aligned} {\hat{\theta }}^w_j := \text{ arg } \text{ max }_{\theta } l(\theta ;D_j^w,E^w). \end{aligned}$$

Classical sampling theory tells us that

$$\begin{aligned} \sqrt{w}\left( {\hat{\theta }}^w_j-\theta _0\right) \mathop {\longrightarrow }\limits ^{\text{ Dist }} N( 0, H), ~~\text{ as } w\rightarrow \infty \end{aligned}$$

for some positive semi definite matrix *H* (see Appendix A for further discussion).

**Fig. 1:**
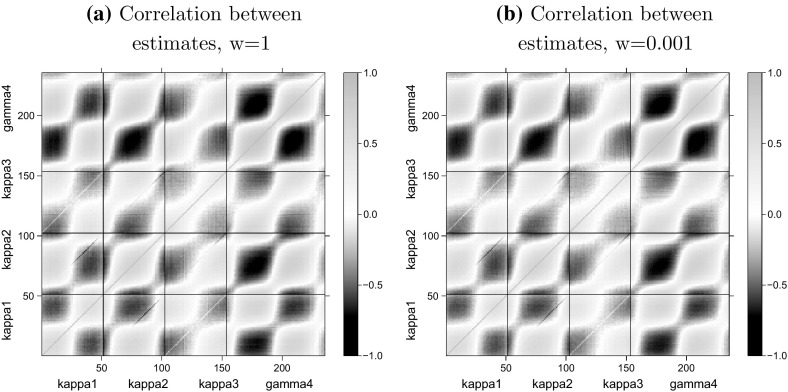
The empirical correlation matrix of the simulated parameter estimates ${\hat{\theta }}^w$ for different values of the population size $w=1$ and $w = 0.001$. The grid lines at 51.5, 102.5 and 153.5 are used to visually separate the parameters $\kappa _t^{(1)}$, $\kappa _t^{(2)}$, $\kappa _t^{(3)}$, $\gamma _c^{(4)}$ from each other in both dimensions. For instance, the *bottom left rectangle* contains the correlations for ${\hat{\kappa }}_{t}^{(1),w}$ for the 51 years from 1960 to 2011

Therefore, we would expect that, even in a finite sample, the co-variance of the distribution of ${\hat{\theta }}^w_j$ is approximately to $w^{-1}H$ and the correlations between different components of ${\hat{\theta }}^w_j$ are approximately independent of the relative population size *w*. Using the simulated sample ${\hat{\theta }}^w_1, \ldots , {\hat{\theta }}^w_N$ we can investigate the finite-sample covariance and correlation matrices of ${\hat{\theta }}^w$. In Fig. 1 we plot a graphical representation of the correlation matrices of ${\hat{\theta }}^w_j$ that we obtain for two values of *w*.

We conclude from Fig. 1 that there are no significant differences between the empirical correlation matrices obtained from different population sizes, as predicted. However, individual components of ${\hat{\theta }}^w$ are not independent from each other as we would expect given the model in (1–3).

**Fig. 2:**
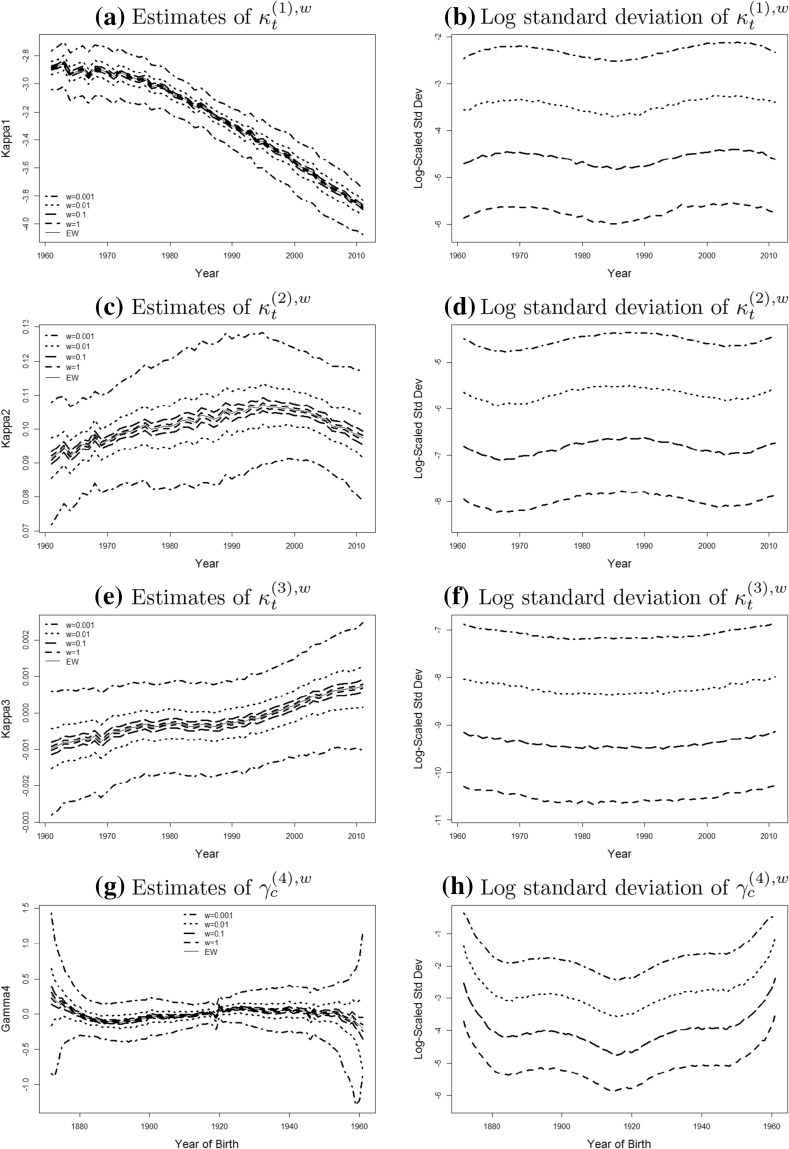
The distribution of MLEs: the mean and confidence interval (*left column*) and the log-scaled standard deviation (*right column*) of the MLEs of $\kappa _{t}^{(1),w}, \kappa _{t}^{(2),w}, \kappa _{t}^{(3),w}, \gamma _{c}^{(4),w}$, with respect to year *t* and year of birth *c* respectively, of populations with $w=1$ (*dashed line*), $w=0.1$ (*long dashed line*), 0.01 (*dotted line*), 0.001 (*dot dashed line*), together with the parameter estimates for the England and Wales population (*solid line*). Note: the upper bound of the CI in the left column is the $95\%$ quantile of the distribution and the lower bound is the $5\%$ quantile

To investigate the finite-sample distribution of the MLE ${\hat{\theta }}^w$ further we plot the empirical mean together with 90% confidence intervals for each of the components of ${\hat{\theta }}^w$ in Fig. 2.

We find for all population sizes considered that the empirical means of the simulated estimates fluctuate around the true parameter values $\theta _0$ (solid line), which indicates that the MLE is approximately unbiased for all considered population sizes. However, the standard deviation of the estimator depends strongly on the size of the population, increasing significantly as the exposures get smaller as can be seen from the width of the confidence intervals.

**Fig. 3:**
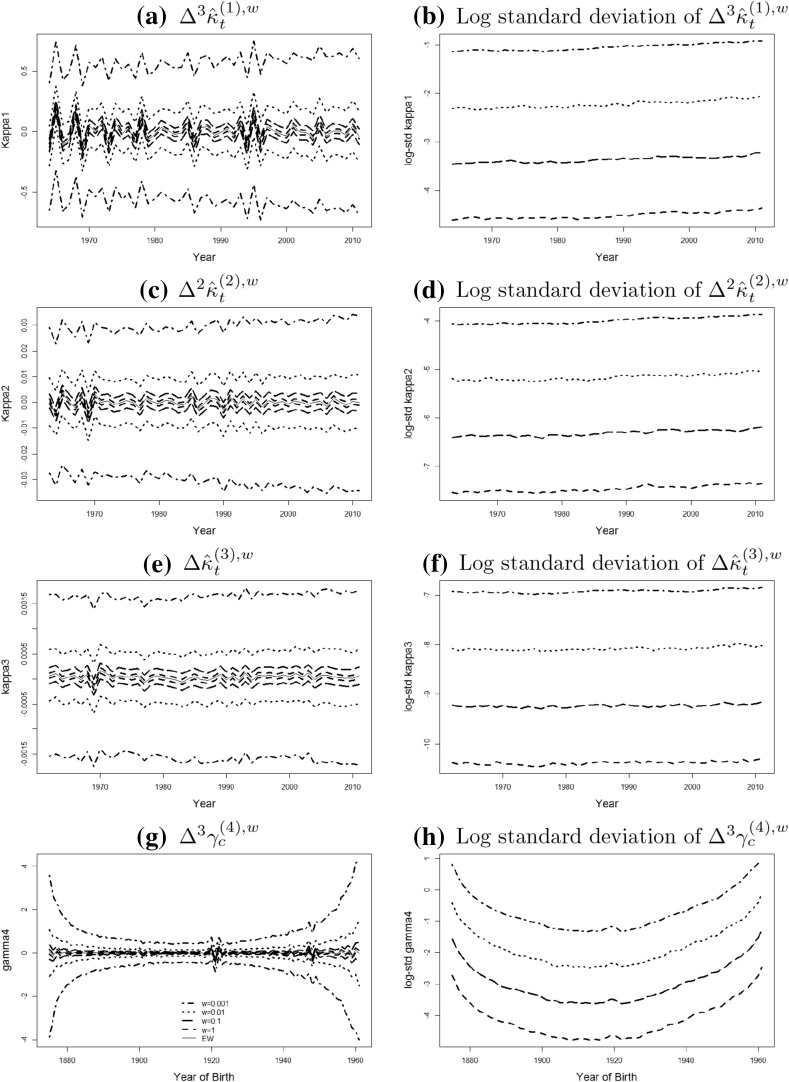
The distribution of $k^{\text{ th }}$ order difference of MLEs: the mean and confidence interval (*left column*) and the log-scaled standard deviation (*right column*) of $\Delta ^3{\hat{\kappa }}_t^{(1),w}$, $\Delta ^2{\hat{\kappa }}_t^{(2),w}$, $\Delta {\hat{\kappa }}_t^{(3),w}$ and $\Delta ^3{\hat{\gamma }}_c^{(4),w}$, with respect to year *t* and year of birth *c* respectively, of populations with $w=1$ (*dashed line*), $w=0.1$ (*long dashed line*), 0.01 (*dotted line*), 0.001 (*dot dashed line*), together with the parameter estimates for the England and Wales population (*solid line*). Note: the upper bound of the CI in the left column is the $95\%$ quantile of the distribution and the lower bound is the $5\%$ quantile

The relative levels of the lines in the graphs on the right hand side of Fig. 2 show that the level of fluctuation increases approximately by a factor $\sqrt{n}$ if the population size is reduced by a factor 1 / *n*, which is consistent with the asymptotic covariance matrix being proportional to 1 / *w*. It also suggests that the variance is generally stable for all the period effects over years, which is not the case for the cohort effect with a wave shaped pattern. We notice that the standard deviation of $\gamma _{c,w}^{(4)}$ widens out considerably at both ends, reflecting the reducing number of observations that we have for the younger and older cohorts. It is worth recalling that the finite-sample distribution of the MLE ${\hat{\theta }}^w$ varies if different sets of constraints are defined in the model system: that is, for a given *w*, the shapes of the various confidence intervals might be different if other identifiability constraints are used. The impact of the identifiability constraints in our study can be removed by calculating the following quantities of the point estimates: $\Delta ^3{\hat{\kappa }}_t^{(1),w}$, $\Delta ^2{\hat{\kappa }}_t^{(2),w}$, $\Delta {\hat{\kappa }}_t^{(3),w}$ and $\Delta ^3{\hat{\gamma }}_c^{(4),w}$ for $w=1, 0.1, 0.01, 0.001$, where $\Delta ^k$ represents the $k^{\text{ th }}$ order difference. The finite-sample distribution of these quantities and the corresponding standard deviation are shown in Fig. 3, where unsurprisingly the right column implies that our conclusion regarding to the proportional relationship between the variance and the population size holds.

## Mortality projections

While fitting the model in (1–3) to observed mortality data only requires the estimation of the period effects $\kappa _t = (\kappa ^{(1)}_t, \kappa ^{(2)}_t, \kappa ^{(3)}_t)'$ and the cohort effect $\gamma ^{(4)}_c$, projecting mortality rates into the future requires a model for values of $\kappa _t$ for $t > t_{n_y}$ where $t_{n_y}$ is the last year for which mortality data are available. Similarly, future values of the cohort effect $\varvec{\gamma ^{(4)}}$ are also required.

The most common approach to obtain future values of $\kappa$ and $\gamma ^{(4)}$ is to consider these parameter vectors as observed trajectories of stochastic processes and fit a parametric time series model to each trajectory. In the following we will fit a three-dimensional random walk to $\kappa _t$ and a stationary AR(1) model to $\gamma ^{(4)}_c$, as in Ref. [8]. We will then discuss the estimation of the parameters of those models based on the values of $\theta _0$ and ${\hat{\theta }}^w_j$ for different values of *w*. This will allow us to investigate the impact of the relative population size *w* on the estimators for the parameters of the $\kappa$ and $\gamma ^{(4)}$ processes.

For the estimation of those parameters and the projections of the period effects and the cohort effect we will consider two approaches. Firstly, we will use a frequentest approach to obtain point estimates of the process parameters ignoring any uncertainty about those estimates. In our further analysis we will follow a Bayesian approach to incorporate parameter uncertainty into our mortality projections.

### Projecting period effects

As mentioned above, we model the period effects $\kappa _t$ as a three-dimensional normal random walk.

8 $$\begin{aligned} \Delta \kappa _t = \mu +L\epsilon _t \end{aligned}$$

where $\Delta \kappa _t = \kappa _t-\kappa _{t-1}$ and the $\epsilon _{t} = (\epsilon _{t}^{(1)}, \epsilon _{t}^{(2)}, \epsilon _{t}^{(3)})'$ are independent random vectors with a multivariate standard normal distribution. The parameter vector $\mu$ is the $3\times 1$ drift vector of the random walk and *L* is the $3\times 3$ Cholesky decomposition of the covariance matrix $V = LL'$.

#### Point estimators

Having generated *N* scenarios for the number of deaths according to (6) and having estimated the parameter vector ${\hat{\theta }}^w_j$ in each scenario as in (7), we can now apply the random walk model to the period effects in $\theta _0$ and ${\hat{\theta }}^w_j$ for every generated scenario *j*. We then apply the usual (i.e. maximum likelihood) point estimators ${\hat{\mu }}^w_j$ and ${\hat{V}}^w_j$ for each simulated scenario of $D^w_j(t,x)$. The estimators for the three components of ${\hat{\mu }}^w_j$ (scenario *j*) are

9 $$\begin{aligned} {\hat{\mu }}^{w}_j(i)=\frac{1}{n_y-1}\sum _{t=t_1+1}^{t_{n_y}}({\hat{\kappa }}_{t,j}^{(i),w}-{\hat{\kappa }}_{t-1,j}^{(i),w}); \qquad i=1,2,3 \end{aligned}$$

and the entries of the estimated $3\times 3$ covariance matrix ${\hat{V}}^w_j$ are

10 $$\begin{aligned} {\hat{V}}^w_j(i,k)=\frac{1}{n_y-1}\sum _{t=t_1+1}^{t_{n_y}}\Big [ \left( \Delta {\hat{\kappa }}_{t,j}^{(i),w}-{\hat{\mu }}^{w}_j(i)\right) \left( \Delta {\hat{\kappa }}_{t,j}^{(k),w}-{\hat{\mu }}^{w}_j(k)\right) \Big ] ;\qquad i,k=1,2,3. \end{aligned}$$

The corresponding estimators for $\mu$ and *V* for the true trajectory $\theta _0$ are defined similarly.

#### Bayesian estimation—parameter uncertainty

As mentioned earlier we model uncertainty about the parameters $\mu$ and *V* by applying a Bayesian approach to estimation. We denote by *p* the density of the prior joint distribution of the two parameters. Assuming that we have no prior knowledge about the true values of $\mu$ and *V*, we use the Jeffreys prior density

$$\begin{aligned} p(\mu ,V)\propto |V|^{-\frac{3}{2}}, \end{aligned}$$

where |*V*| is the determinant of *V* (see for example, [16]). Using this prior distribution in each scenario *j*, the posterior distribution is given by the inverse Wishart distribution for *V* and a multivariate normal distribution for $\mu$, that is,

11 $$\begin{aligned} \left( {\tilde{V}}^w_j\right) ^{-1}| \Delta {\hat{\kappa }}^w_j\sim ; {} {\text {Wishart}}(n_y-2,(n_y-1)^{-1}({\hat{V}}^w_j)^{-1}) \end{aligned}$$

12 $$\begin{aligned} {\tilde{\mu }}^w_j|{\tilde{V}}^w_j,\Delta {\hat{\kappa }}^w_j\sim & {} N({\hat{\mu }}^w_j,(n_y-1)^{-1}{\tilde{V}}^w_j) \end{aligned}$$

where ${\hat{\mu }}^w_j$ and ${\hat{V}}_{j}^w$ are the estimates obtained from ${\hat{\theta }}^w_j$ as defined in (9) and (10).

#### Empirical comparison

**Fig. 4:**
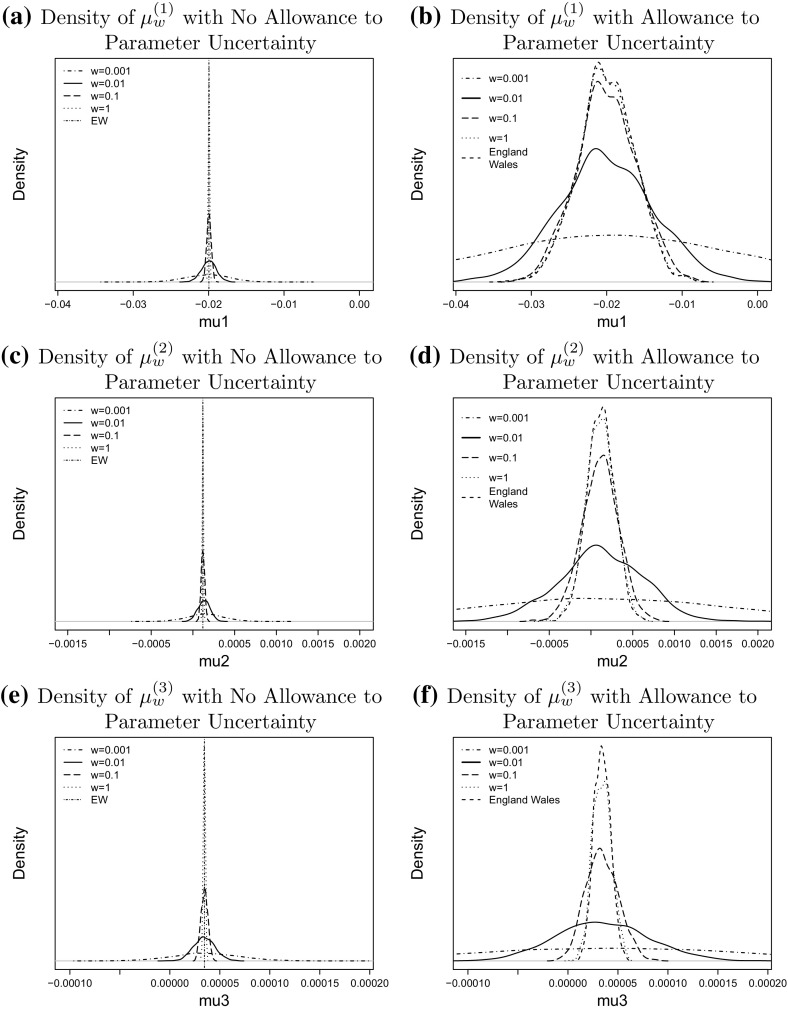
The impact of population size on the distribution of the random walk drift, from population of $w=1$ (*dotted line*), $w=0.1$ (*long-dashed line*), $w=0.01$ (*solid line*), $w=0.001$ (*dot dashed line*) and England and Wales (*vertical line*). The *left column* is the density of drift without allowance to the parameter uncertainty; the right column is the density of drift with allowance to the parameter uncertainty

For our empirical study we simulate $N = 1000$ scenarios for different values of *w* and plot the empirical density of the point estimator ${\hat{\mu }}^w$ in (9) based on the sample ${\hat{\mu }}^w_1, \ldots , {\hat{\mu }}^w_N$ on the left hand side of Fig. 4. To incorporate parameter uncertainty we draw a further sample of size $M = 100$ from the posterior distribution of ${\tilde{\mu }}^w_j$ in (12) in each scenario $j = 1, \ldots , N$. The empirical density of ${\tilde{\mu }}^w_j$ from these $N \times M$ realisations is shown on the right hand side of Fig. 4.

By comparing the densities in the two columns of that figure we observe that the additional parameter uncertainty increases the variance of the empirical distributions of the drift estimators. This can be explained by investigating the source of uncertainty to the drift. The variation to the point estimator ${\hat{\mu }}^w(i)$ with no allowance for parameter uncertainty comes from the Poisson noise in the number of deaths from the bootstrap simulations, while the variance of the Bayesian estimator ${\tilde{\mu }}^w(i)$ with allowance for extra parameter uncertainty also includes the uncertainty (Eq. 12) from the posterior distribution given the Poisson noise.

**Fig. 5:**
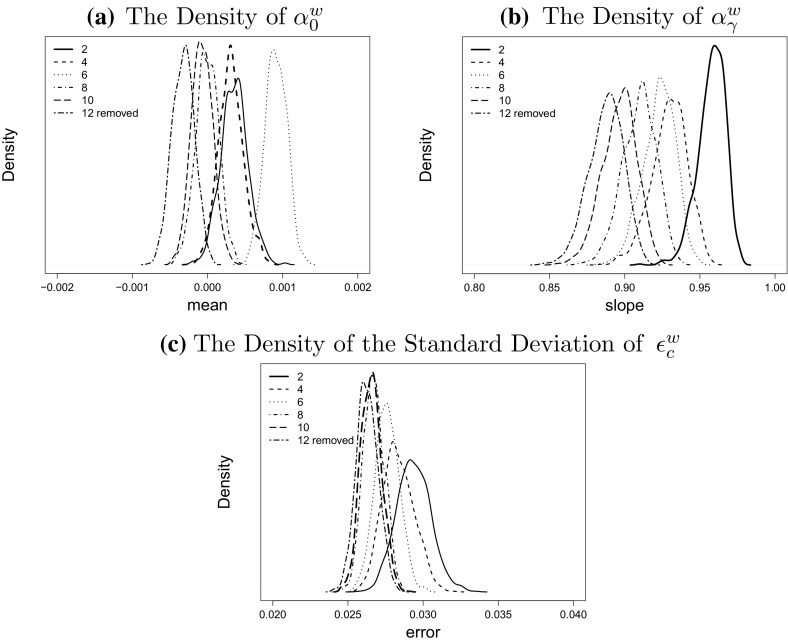
The effect of removing the cohort effects of short cohorts on the distribution of the parameter estimators of the *AR*(1) model for $w=1$. We investigate the distribution of parameter estimates when the first and last 1, 2, 3, 4, 5, 6 cohorts are removed

We also find in Fig. 4 that the size of a population affects the uncertainty about the drift vector $\mu$. The variance of the empirical finite sample distribution of both estimators, ${\hat{\mu }}$ and ${\tilde{\mu }}$ decreases significantly when the population size increases, although the difference between $w=1$ and $w=0.01$ is rather small as is particularly obvious for the Bayesian estimator ${\tilde{\mu }}$ Fig. 5

However, for smaller values of *w* we find that the population size has a much more pronounced effect on the variance. For example, the range of likely values of ${\tilde{\mu }}^{0.001}$ is significantly wider than the range of values of ${\tilde{\mu }}^{0.1}$ and ${\tilde{\mu }}^1$ reflecting the uncertainty about $\mu ^w$ that we have already observed in Fig. 2 top left. The same argument applies to the point estimators ${\hat{\mu }}$.

To investigate parameter uncertainty further we calculate the standard deviations for the distributions of ${\hat{\mu }}$ and ${\tilde{\mu }}$ in Fig. 4. Those standard deviations are shown in Table 2. We observe that the standard deviation of the point estimator ${\hat{\mu }}$ is increased approximately by a factor $\sqrt{10}$ if the population size is reduced by a factor 10. The situation becomes more complicated when for the Bayesian estimator ${\tilde{\mu }}$ since the variance of the posterior distribution affects the finite sample variance of the estimator. There is no obvious proportional relationship between population size and variation, which suggests that the size of the population is not the only determinant of the variance of ${\tilde{\mu }}$.

**Table 2:** The finite sample standard deviation of ${\hat{\mu }}$ and ${\tilde{\mu }}$

		$i = 1$	$i = 2$	$i = 3$
Point estimator ${\hat{\mu }}^w(i)$	w = 1	0.0000966	0.0000071	0.00000113
	w = 0.1	0.0003050	0.0000217	0.00000343
	w = 0.01	0.0009777	0.0000727	0.00001068
	w = 0.001	0.0028787	0.0002206	0.00003387
Bayesian estimator ${\tilde{\mu }}^w(i)$	w = 1	0.00369	0.000173	0.00000936
	w = 0.1	0.00396	0.000222	0.0000162
	w = 0.01	0.00620	0.000505	0.0000458
	w = 0.001	0.01689	0.001566	0.0001478

To investigate the impact of the relative population size *w* and the inclusion of parameter uncertainty on the empirical distribution of the estimated co-variance matrix *V* of the random walk in (8) we compare the empirical means of ${\hat{V}}$ and ${\tilde{V}}$ obtained for different values of *w*. The means of the estimated co-variance matrix ${\hat{V}}$ are:

$$\begin{aligned} E[{\hat{V}}^1]= & {} \left( \begin{array}{ccc} 6.82\times 10^{-4}&{} 2.12\times 10^{-5}&{} 5.42\times 10^{-7}\\ 2.12\times 10^{-5}&{} 1.41\times 10^{-6}&{} 2.99\times 10^{-8}\\ 5.42\times 10^{-7}&{} 2.99\times 10^{-8}&{} 4.30\times 10^{-9}\\ \end{array}\right) \\ E[{\hat{V}}^{0.01}]= & {} \left( \begin{array}{ccc} 18.7\times 10^{-4}&{} -1.52\times 10^{-5}&{} 4.61\times 10^{-6}\\ -1.52\times 10^{-5}&{} 1.25\times 10^{-5}&{} -1.89\times 10^{-7}\\ 4.61\times 10^{-6}&{} -1.89\times 10^{-7}&{} 0.99\times 10^{-7} \end{array}\right) \end{aligned}$$

and the mean values of the Bayesian estimator ${\tilde{V}}$ are

$$\begin{aligned} E[{\tilde{V}}^1]= & {} \left( \begin{array}{ccc} 7.58\times 10^{-4}&{} 2.37\times 10^{-5}&{} 6.06\times 10^{-7}\\ 2.37\times 10^{-5}&{} 1.58\times 10^{-6}&{} 3.35\times 10^{-8}\\ 6.06\times 10^{-7}&{} 3.35\times 10^{-8}&{} 4.79\times 10^{-9}\\ \end{array}\right) \\ E[{\tilde{V}}^{0.01}]= & {} \left( \begin{array}{ccc} 20.90\times 10^{-4}&{} -1.74\times 10^{-5}&{} 5.11\times 10^{-6}\\ -1.74\times 10^{-5}&{} 1.39\times 10^{-5}&{} -2.10\times 10^{-7}\\ 5.11\times 10^{-6}&{} -2.10\times 10^{-7}&{} 1.11\times 10^{-7} \end{array}\right) . \end{aligned}$$

The corresponding estimated covariance matrices, $V^{EW}$, for England and Wales based on the single sample paths of $\kappa _t$ and $\gamma _c$ and the mean of Bayesian estimator are

$$\begin{aligned}{rCl} {\hat{V}}^{EW}= & {} \left( \begin{array}{ccc} 6.70\times 10^{-4}&{} 2.16\times 10^{-5}&{} 4.94\times 10^{-7}\\ 2.16\times 10^{-5}&{} 1.31\times 10^{-6}&{} 3.18\times 10^{-8}\\ 4.94\times 10^{-7}&{} 3.18\times 10^{-8}&{} 3.30\times 10^{-9} \end{array}\right) \\ E[{\tilde{V}}^{EW}]= & {} \left( \begin{array}{ccc} 5.49\times 10^{-4}&{} 1.80\times 10^{-5}&{} 1.05\times 10^{-7}\\ 1.80\times 10^{-5}&{} 1.07\times 10^{-6}&{} 2.19\times 10^{-8}\\ 1.05\times 10^{-7}&{} 2.19\times 10^{-8}&{} 3.06\times 10^{-9} \end{array}\right) \end{aligned}$$

Comparing the mean values of ${\hat{V}}$ and ${\tilde{V}}$ with the estimates obtained from the England and Wales data we find significant differences in the estimated covariances. In particular, for smaller populations (e.g. *w*=0.01) sampling variation pushes up significantly estimates of the covariance matrix. In addition, sampling variation also widens the distribution of *V* around these mean values for smaller values of *w*. On the other hand, for a given value of *w*, the inclusion of full Bayesian parameter uncertainty moving from ${\hat{V}}$ to ${\tilde{V}}$ has rather less of an impact.

Finally, the projected parameters based on the Bayesian estimates ${\tilde{\mu }}$ and ${\tilde{V}}$ are shown in Fig. 7. As we expected, the prediction intervals reflecting the uncertainty about future values of the period effects are very wide for small populations. The plots also suggest that the means of the co-variances are right biased compared to the estimate for England and Wales. The variance of projection for all the populations are much higher than the estimates, due to the additional normal randomness added in the forecasting model by simulating the sample paths for $\kappa$ and $\gamma ^{(4)}$. However, the left column shows that there is no obvious proportional relationship between the population size and projection variance. By investigating the mean co-variance matrices, we find that the increase of $E[V_{3,3}^w]$ from $w=0.01$ to $w=1$ is of the highest among the three period effects, which suggests that the standard deviation of projection for $\kappa _t^{(1)}$ and $\kappa _t^{(2)}$ is not as sensitive as $\kappa _t^{(3)}$ to the change of population size.

### Projecting the cohort effect

As mentioned earlier we fit an AR(1) model to the cohort effect. We will not investigate how additional parameter uncertainty influences mortality projections, but will only use point estimates for the parameters in the AR(1) model. To be precise, our model is given by

13 $$\begin{aligned} \gamma _{c+1}^{(4)}= \;\alpha _0+\alpha _{\gamma } \left( \gamma _c^{(4)}-\alpha _0\right) +\epsilon _{c+1} \end{aligned}$$

Figure 2 shows that the variance of the estimated cohort effect is very large for the very early and very late years of birth, in particular, for $w=0.01$ and 0.001. This is a consequence of the very few observations available for those cohorts. We therefore remove the cohorts with six or less observations. Cohorts are removed equally from the beginning and the end.

However, removing short cohorts could significantly influence the estimated values of the parameters. To investigate the effect of removing short cohorts in more detail we plot the empirical densities for the parameters in (13) based on the estimated parameters in each simulated scenario *j* for $w=1$. We find that the distribution of ${\hat{\alpha }}_0$ is not significantly affected by removing cohorts which is also the case for the estimated variance of $\epsilon _c$ when more than 4 cohorts are removed in total. In contrast, ${\hat{\alpha }}_{\gamma }$ is shifted to the left as more cohorts are removed. Further we notice that the variance of the estimators for all three parameters stays approximately unchanged regardless of how many cohorts are removed.

**Fig. 6:**
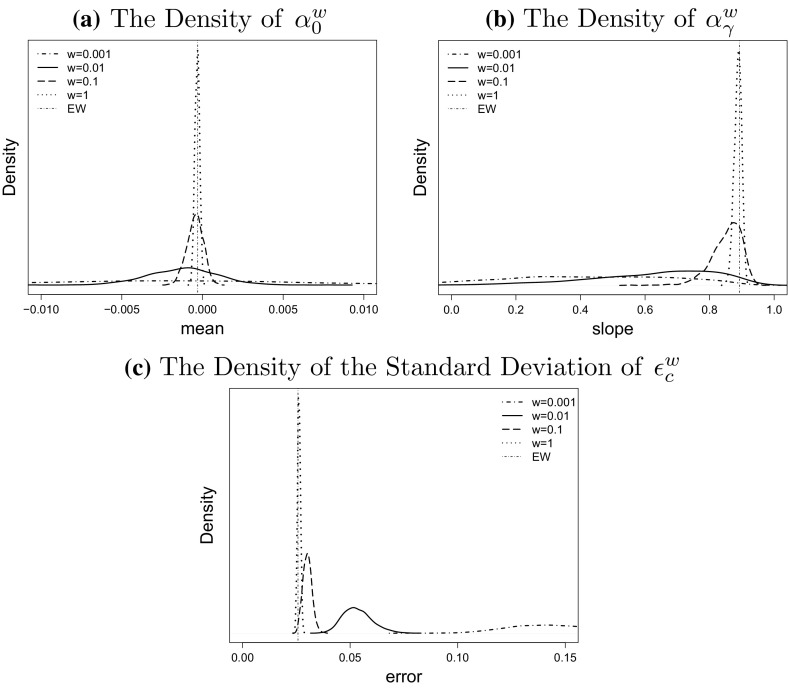
The comparison of the distribution of the parameter estimates of the AR(1) model between the constructed populations* w* = 1 (*dotted line*),* w* = 0:1 (*long dashed*), *w* = 0:01 (*solid*),* w* = 0:001 (*dot dashed*) and England and Wales (*vertical line*)

After having removed cohorts with six or less observations from the data, we fit the AR(1) model in (13) to the rest of the cohort effects. The resulting density of the parameter estimates of the model are shown in Fig. 6. All of the parameter estimates and the standard deviation of error terms appear to be biased relative to the estimate for England and Wales, regardless of the size of population. However, we find that reducing the population size will greatly increase the mean bias as well as the uncertainty.

We now forecast the cohort effect from ${\hat{\gamma }}_{t_{n_y}-x_1-6}^{(4),w}= {\hat{\gamma }}_{1955}^{(4),w}$ instead of ${\hat{\gamma }}_{1961}^{(4),w}$ and the result is shown in Fig. 6d. The variation in the projected cohort effects for the years 1956 to 1961 now comes from the Poisson and Normal randomness, which is not as great as variation at the two tails of the estimates observed in Fig. 2 where no cohorts have been removed. Within the sample, the confidence intervals are narrower for cohorts with greater numbers of observed years (ranging from 7 to 40) and greater numbers of deaths since variance is reduced by having more number of observations.

### Projected mortality rates

Based on the projected period and cohort effects we can now turn to the projection of mortality rates using our model in (1)–(3). Figure 8 shows the twenty-year forward projections of mortality rates at ages 65 and 85. We compare the predicted rates with and without the allowance for parameter uncertainty for all the constructed populations with the projections based on the England and Wales data. Unsurprisingly, the uncertainty about future mortality rates increases as the forecast horizon increases. The other two factors which significantly influence the projection uncertainty are age and population size.

Reducing the population size results in greater uncertainty about mortality forecasts for both ages. For example, the uncertainty is much greater for the smaller populations ($w=0.01, 0.001$) at both ages 65 and 85. This means that there is considerable uncertainty about future mortality scenarios for a relatively small pension scheme with significant implications for the risk management of such a scheme.

Comparing parts (a) and (b) of Fig. 8 we find that the inclusion of parameter uncertainty for the drift parameter $\mu$ adds further uncertainty about the projected mortality rates. This reflects the additional randomness from not having a sufficiently long period of observed rates. We notice that the difference of variance between including and excluding parameter uncertainty increases as time increases. Thus parameter uncertainty becomes much less important when only relatively short forecast horizons are considered. Similar results can be found in Fig. 6 of [6] which shows the log scaled variance of both, with and without parameter uncertainty, for the survival index. Our findings are also in line with results obtained by [20] who have found that the uncertainty about the drift of the period effect in a Lee-Carter model has little impact on the uncertainty of short term projections while it significantly affects the uncertainty of long-term projections. This supports our conclusion that the differences in the variances are tiny when the projection horizon *t* is very small, and become more significant for long term projection. We notice that for age 65 the intervals are not smooth in some years due to the cohort effect.

**Fig. 7:**
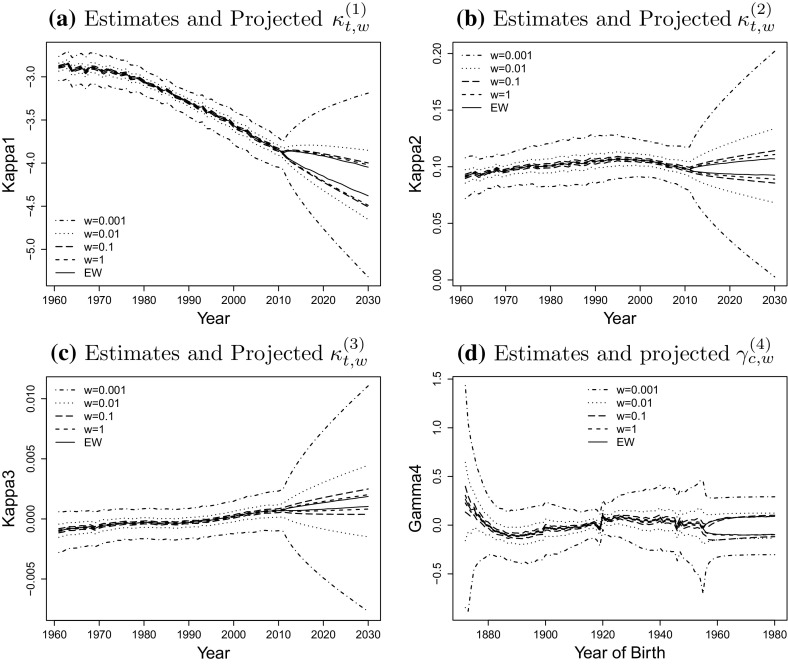
The comparison of twenty-year forward projection of $\kappa _{t,w}^{(1)}, \kappa _{t,w}^{(2)}, \kappa _{t,w}^{(3)}, \gamma _{c,w}^{(4)}$, of weight $w=1, 0.1, 0.01, 0.001$ with England and Wales. Note: We forecast the cohort effect from the last sixth cohort instead of the very last one due to the cohort removal. The upper bound of the CI is the $95\%$ quantile of the distribution and lower bound is the $5\%$ quantile. Parameter uncertainty is allowed in the projection

**Fig. 8:**
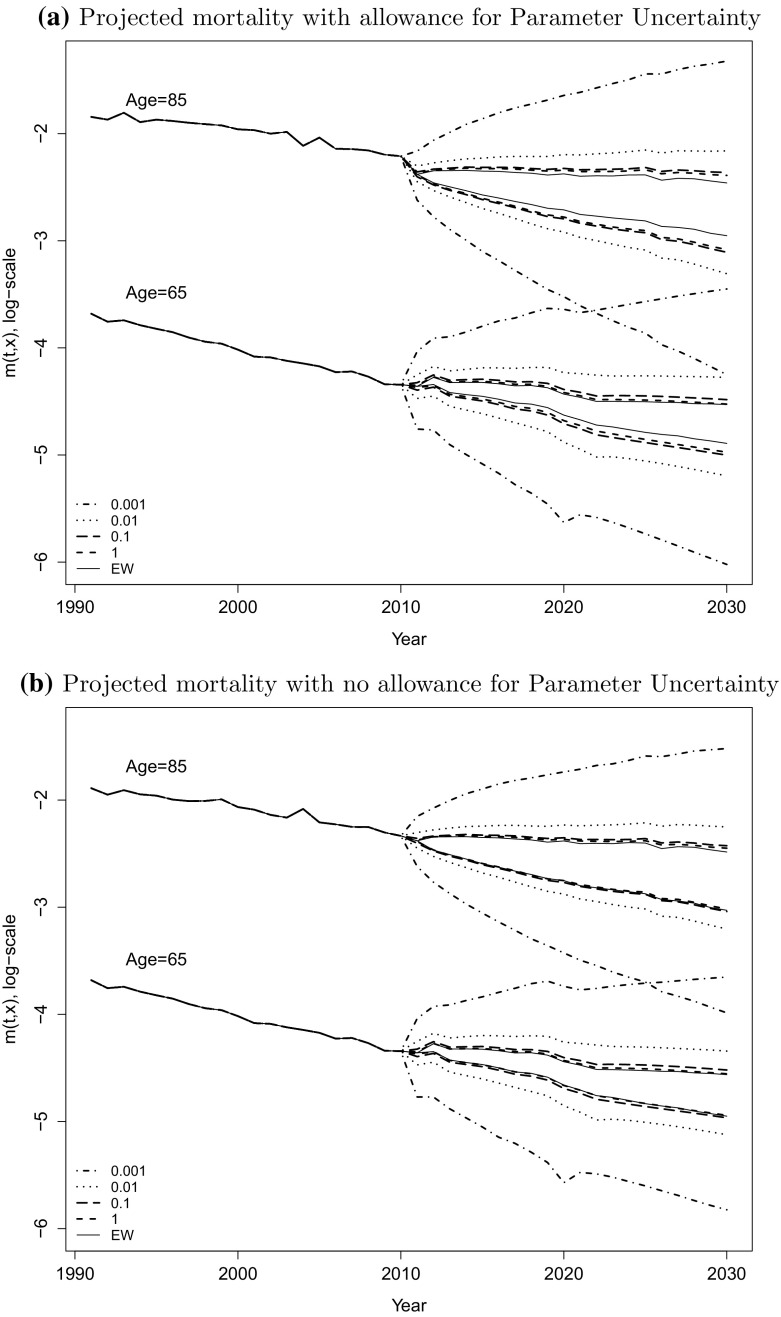
The log-scaled 90% prediction intervals of twenty-year forward mortality rate projections with (*upper plot*, **a**) and without (*lower plot*, **b**) allowance for parameter uncertainty at ages 65 and 85, for population size $w=1$ (*dashed line*), 0.1 (*long dashed line*), 0.01 (*dotted*), 0.001 (dot dashed line) and England and Wales (*solid line*). Note that the *solid line* at the* left end* is the estimated mortality rate of the England and Wales population, with length of 20 years.* The upper bound *of the prediction interval is the 95$\%$ quantile of the distribution and* lower bound* is the 5$\%$ quantile

**Fig. 9:**
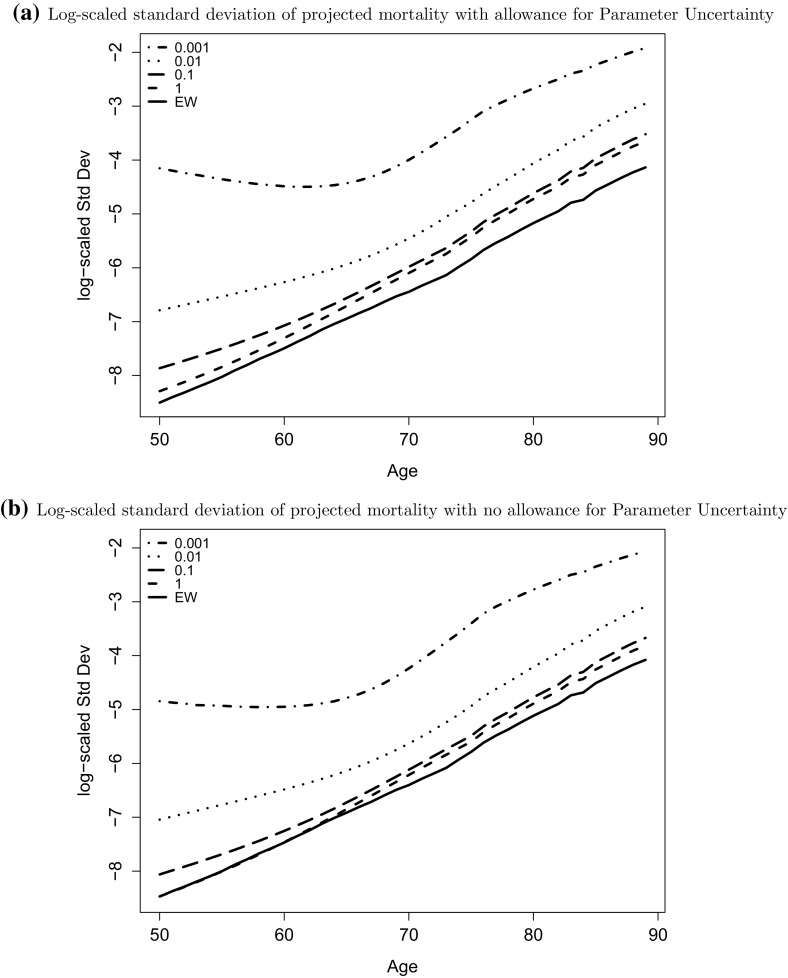
The log-scaled standard deviation of projected mortality rates with (*upper plot*) and without (*lower plot*) allowance for parameter uncertainty in year 2030 with respect to age for population size $w = 1$ (*dashed line*), 0.1 (*long dashed line*), 0.01 (*dotted*), 0.001 (*dot dashed line*) and England and Wales (*solid line*)

We also notice that age seems to affect the amount of uncertainty around the central projection differently in small and large populations. To illustrate this further we consider the standard deviation of projected mortality rates as a function of age for a fixed projection horizon. Figure 9 shows the log-scaled standard deviation of the projected mortality rates in the calendar year 2030 with respect to age. We find in this figure that the variance is an increasing function of age if the population size is rather large. In contrast, we find for the smallest population ($w=0.001$) that the variance only starts to increase from about age 70 while it is constant or slightly decreasing for younger ages. As we found in Fig. 8, at age 65 (and also at age 85), the three largest populations have prediction intervals which are of similar width. However, Fig. 9 shows that the much wider prediction intervals for the two smaller populations seem to be less affected by age with the relative increase in the standard deviation from age 65 to 85 being smaller than for the large populations. We are also interested in how much of the forecast variation is due to the impact of sampling variation and parameter uncertainty on the covariance matrix, *V*, and the drift, $\mu$. To investigate this, we consider four experiments outlined below. Note that we still projected the cohort effect, given the point estimates for population *w* with the method introduced in Sect. 4.2 and we sample from the empirical distribution of ${\hat{\mu }}$ and ${\hat{V}}$ without considering the Bayesian posterior.

1. Project mortality rates for each constructed population, while fixing the parameters $\mu$ and *V* of the random walk to the estimates obtained from the England and Wales data.
2. Project mortality rates for each constructed population, while fixing only the drift $\mu$ to the corresponding EW estimates and sample realisations of ${\hat{V}}$ from its empirical distribution.
3. Project mortality rates for each constructed population, while fixing only the variance matrix *V* to the corresponding EW estimates and sample the drift parameter from the empirical distribution of ${\hat{\mu }}$.
4. Project mortality rates when both *V* and $\mu$ are samples from the empirical distribution of ${\hat{V}}$ and ${\hat{\mu }}$.

**Fig. 10:**
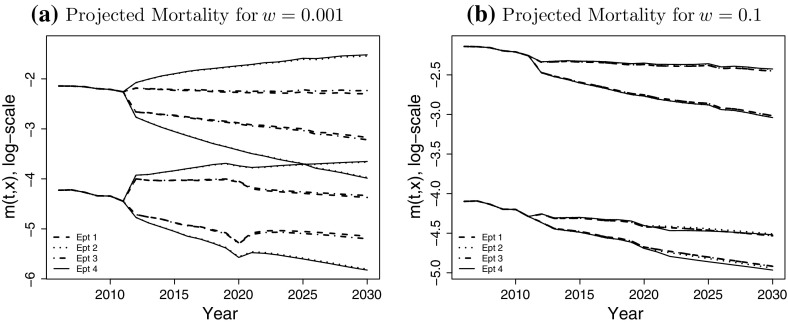
The projected mortality rates at age 65 and 85 for population sizes $w=0.001$ (*left*), $w=0.1$ (*right*) for the four experiments outlined in Table 4.3. The upper bound of the prediction interval is the 95% quantile of the distribution and the* lower bound* is the 5% quantile. Note that the* solid line* at the* left end* is the true mortality rate of the England and Wales population, up to year 2011

The results are shown in Fig. 10. We find that fixing parameters has a significant effect on mortality forecasting when populations are very small (w = 0.001) in Fig. 10a. We can see that the widths of prediction intervals for our experiments 1 and 3 are much narrower than for experiments 2 and 4, and the difference of variance is greater for long term projections. The major difference between these two scenarios is that we fix the co-variance matrix *V* to its estimate obtained from England and Wales data in experiments 1 and 3. Thus we conclude that a major source of uncertainty for our mortality forecasts comes from the bias in the estimated covariance matrix for small populations.

### Summary

To summarise, forecasts levels of uncertainty in future mortality are biased upwards for two reasons. First, and most obvious, the Poisson noise in the data biases up estimates of the random walk covariance matrix to a significant extent (Fig. 10). Second, when we include a Bayesian analysis of parameter uncertainty, uncertainty in the random walk drift resulting from observation over a relatively small number of years is pushed up by the small population bias in the covariance matrix, *V*. This has its greatest impact in longer term forecasts, and less impact in the short term.

## Likelihood ratio test for systematic parameter difference

We have seen that the size of a population has a substantial impact on the level of uncertainty about the parameters of the model in (1–3) when this model is fitted to the population’s mortality data. This raises the question whether the estimated period and cohort effects in $\theta = (\kappa _t^{(1)},\kappa _t^{(2)},\kappa _t^{(3)},\gamma _c^{(4)})$ for a small a population are significantly different from those in a given, typically much larger, reference population. To address this question we apply a likelihood ratio test to test for significant deviations of estimated parameters from a given null hypothesis using the maximum likelihood estimator ${\hat{\theta }}^w_j$ defined in (7) for simulated mortality data $D^w_j$ as in (6). We are particularly interested in the finite sample distribution of the test statistic as compared to its asymptotic distribution. As in Sect. 3 we will use simulated deaths scenarios to investigate the finite sample distribution and the power of the likelihood ratio test (LRT) applied to mortality data. We will start with a short review of the LRT.

### Review of likelihood ratio test

The LRT used in this study follows the generalized form of the LRT as defined in Ref. [19]. For a random variable *X* with a distribution that depends on a parameter vector $\theta$, the likelihood function is defined as usual:

$$\begin{aligned} L(x|\theta ):=\prod _{i=1}^{n}f_i(x_i|\theta ), \end{aligned}$$

where $f_i(.|\theta )$ is the probability density function of $X_i$ given the parameter vector $\theta$. We assume that $\theta :=(\theta _r, \theta _s)$ is a vector of $r+s$ parameters. The null hypothesis and alternative for the LRT concern only the parameters in $\theta _r$, that is,

14 $$\begin{aligned} H_0: \theta _r=\theta _{r0};~H_1: \theta _r\ne \theta _{r0}. \end{aligned}$$

In order to calculate the test statistic, we first find the MLE of $(\theta _r,\theta _s)$, which leads to the unconditional maximum of the likelihood function

15 $$\begin{aligned} {\hat{\theta }}:= ({\hat{\theta }}_r, {\hat{\theta }}_s) := \text{ arg } \text{ max}_{(\theta _r, \theta _s)} L(x~|~\theta _r,\theta _s). \end{aligned}$$

We then find the MLE of $\theta _s$ assuming that the null hypothesis is fulfilled, that is,

16 $$\begin{aligned} {\tilde{\theta }}_s := \text{ arg } \text{ max}_{\theta _s} L(x~|~\theta _{r0},\theta _s). \end{aligned}$$

In general ${\tilde{\theta }}_s\equiv {\tilde{\theta }}_s(\theta _{r0}) \ne {\hat{\theta }}_s$. We use the notation ${\tilde{\theta }}_s(\theta _{r0})$ to emphasis that ${\tilde{\theta }}_s$ is conditional on the value of $\theta _{r0}$.

We now define the test statistic in the usual way:

17 $$\begin{aligned} \Gamma :=-2{\text{log}}\frac{L(x~|~\theta _{r0},{\tilde{\theta}}_s)}{L(x~|~{\hat{\theta}}_r,{\hat{\theta}}_s)}. \end{aligned}$$

[33] proved that when $H_0$ holds, $\Gamma$ asymptotically follows a central $\chi ^2$ distribution with *r* degrees of freedom. From the central limit theorem, it follows that the $\chi _r^2$ distribution can be approximated by a normal distribution with mean *r*, given *r* is sufficiently large.^2^ Thus we expect that the distribution of $\Gamma$ should approximately be symmetric around *r*.

Before we start testing our null hypothesis, it is worth considering the testability of the hypothesis.^3^ In our approach the constraints in Equation (4) in Sect. 2 are part of the model and therefore the effective number of parameters that are identifiable is the total number of parameters reduced by the number of constraints. In this paper, we formulate the constraints in terms of the cohort effect $\gamma$ since we will in particular consider the case $\theta _{r}=\gamma$ in our empirical study. If the test is about one of the period effects we could reformulate the constraints in terms of that period effect (strictly, therefore, a different model). In that way, the constraints are always fulfilled under $H_0$. In short, the constraints should be chosen such that the null hypothesis fulfils the constraints. In other words, we are testing the null hypothesis that the mortality experience is generated by mortality rates that follow model M7 with the constraints in Equation (4) and $\theta _{r}=\theta _{r_0}$.

In the reminder of this section we will consider a null hypothesis about the entire parameter vector $\theta$ setting $s=0$. In Sect. 7 we will then consider a null hypothesis about the cohort effect $\gamma$ only, that is $s>0$.

### Finite sample distribution of LRT

As mentioned above, we now consider a test for systematic parameter differences involving all period effects and the cohort effect, that is, $s=0$ and $\theta =\theta _r = (\kappa _t^{(1)},\kappa _t^{(2)},\kappa _t^{(3)},\gamma _c^{(4)})$. The null hypothesis and alternative are given in (14), and the LRT statistic is defined in (17) which simplifies to

18 $$\begin{aligned} \Gamma =-2{\text {log}}\frac{L(x~|~\theta _{r0})}{L(x~|~{\hat{\theta }}_r)} \end{aligned}$$

since $$s=0$$.

As in Sect. 3, we choose the male population in England and Wales as our base case and set $\theta _0 = {\hat{\theta }}^{\text{ EW }}$.

To investigate the finite sample properties of the LRT in small populations we apply a parametric bootstrap procedure in which we simulate *N* mortality scenarios, estimate the parameter vector $\theta$ as in Sect. 3 and apply the LRT in each scenario. More precisely we use the following steps to find a bootstrap approximation of the finite sample distribution of $\Gamma$: for different values of *w* and for each scenario $j = 1 \ldots N$ we

1. simulate $D^w_j$ as in (6),
2. find the estimate ${\hat{\theta }}^w_j$ as in (7),
3. calculate the realisation of the LRT statistic $\Gamma ^w_j$ as in (18) and
4. calculate the *p*-value $P^w_j$ based on the asymptotic $\chi ^2$-distribution as $P^w_j = P[X > \Gamma ^w_j]$ where *X* is has $\chi ^2$-distribution with $\alpha$ degrees of freedom.

The degrees of freedom of the $\chi ^2$ distribution in step 4 should be the effective number of parameters denoted by $\alpha$, which is the total number of parameters *r* less the number of constraints, that is

$$\begin{aligned} \alpha = 3n_{y}+n_{c}-3 \end{aligned}$$

where $n_{y}$ is the number of years, and $n_{c}=n_y + n_a -1$ is total number of cohorts in a given dataset without removing short cohorts. In our case, $n_{y}=51$, $n_{c}=51 + 40 -1 = 90$, hence $\alpha =240$. After applying the parametric bootstrap method we can generate the distribution of the test statistic. We expect that the distribution of $\Gamma ^w$ should be approximately symmetric around 240.

**Fig. 11:**
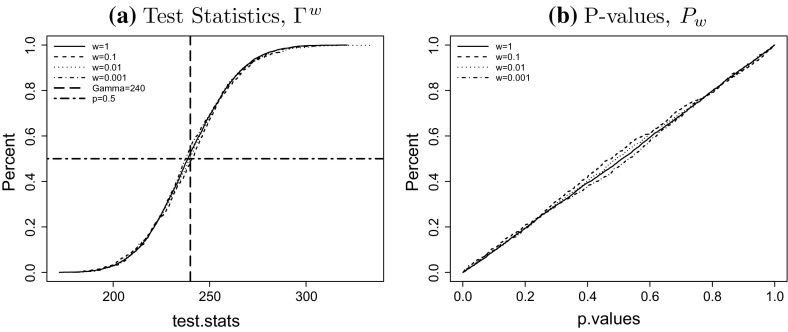
Likelihood ratio test: **a** empirical CDF’s of test statistics for sample size $N=1000$. **b** empirical CDF’s of asymptotic p-values. Results shown for populations of $w=1$ (*solid line*), $w=0.1$ (*dashed line*), $w=0.01$ (*dotted line*) and $w=0.001$ (*dot dashed line*). The mean of the asymptotic $\chi _{240}^2$ distribution is also shown as the *vertical dashed line* in plot (**a**)

For any population size *w* we can now find the empirical distribution function of $\Gamma ^W$ based on the sample $\Gamma ^w_1, \ldots , \Gamma ^w_N$. Furthermore, if the asymptotic $\chi ^2$ approximation is accurate, the *p*-values $P^w_1,\ldots ,P^w_N$ should be independent and uniformly distributed on [0, 1]. The cumulative distribution of the test statistic $\Gamma ^w$ and the p-values $P^w$ for all considered population sizes *w* are shown in Fig. 11 for $N=1000$. Figure 11a shows that the empirical distribution of $\Gamma ^w$ is indeed centred around $\alpha =240$. We also observe in Fig. 11b that the cumulative distribution function of the *p*-values resembles the distribution function of the uniform distribution on [0, 1]. Both results indicate that the $\chi ^2$ approximation for the distribution of $\Gamma ^w$ under the null hypothesis is very good for all values of *w* considered.

### Power of the likelihood ratio test

In the last section, we carried out the likelihood ratio test for the parameter difference and found that the $\chi ^2$ approximation does not fail to capture the feature of the test statistic $\Gamma ^{w}$ when $H_0$ holds. We will now investigate how the population size affects the power of LRT. In general, the power of a binary hypothesis is the probability of correctly accepting the alternative hypothesis when it is true.^4^

As usual the power of a test is defined as

$$\begin{aligned} \text {Prob}~(\text {Reject}\,H_0\,|\,H_1\,\text {is\,True}). \end{aligned}$$

To evaluate the power of the LRT with a parametric bootstrap procedure similar to the one used in the previous section we need to generate scenarios under the alternative. So far we have considered a very general alternative $\theta _r \ne \theta _{r0}$. We will now need to specify this alternative further. To this end we define four alternative models and investigate the power assuming that the “true” data generating model is one of those alternatives. The four models we consider for the alternative shift or scale one of the period effects or the cohort effect estimated from the England and Wales data.

More specifically, the alternatives we consider are:

- $$\theta ^{(1)}=({\hat{\kappa }}_0^{(1)}+\lambda ,{\hat{\kappa }}_0^{(2)},{\hat{\kappa }}_0^{(3)},{\hat{\gamma }}_0^{(4)})$$
- $$\theta ^{(2)}=({\hat{\kappa }}_0^{(1)}, {\hat{\kappa }}_0^{(2)}+\lambda ,{\hat{\kappa }}_0^{(3)},{\hat{\gamma }}_0^{(4)})$$
- $$\theta ^{(3)}=({\hat{\kappa }}_0^{(1)}, {\hat{\kappa }}_0^{(2)},{\hat{\kappa }}_0^{(3)}+\lambda ,{\hat{\gamma }}_0^{(4)})$$
- $$\theta ^{(4)}=({\hat{\kappa }}_0^{(1)}, {\hat{\kappa }}_0^{(2)},{\hat{\kappa }}_0^{(3)},\lambda {\hat{\gamma }}_0^{(4)})$$

We then evaluate the power of the LRT against each of those alternatives with different values of $\lambda$. Note that we scaled the cohort effect by $\lambda$ units instead of shifting it since shifting the cohort effect would result in the same fitted mortality rates as shifting $\kappa _t^{(1)}$ in $\theta ^{(1)}$. We note that a more general alternative could be considered by allowing for combinations of the above. However, we wish to focus on the impact of misspecifying individual parameters and the power of the test to detect those misspecification.

We can now proceed as in the previous section with simulating death counts and then apply the LRT for different alternatives and different values of $\lambda$. We define $D_{j}^{w,(i)}(t,x)$ to be the simulated deaths in scenario $j=1 \ldots N$ for the population of size $wE_0(t,x)$ using the parameter $\theta ^{(i)}$ in our model, that is,

$$\begin{aligned} D_{j}^{w,(i)}(t,x) \sim \text {Pois}(m(\theta ^{(i)},t,x)wE_0(t,x)) \end{aligned}$$

for any $i=1,2,3,4$, where *m* is defined in (2) and (3). Note that death counts also depend on $\lambda$.

Using the simulated death counts $D_{j}^{w,(i)}$ we obtain the MLE ${\hat{\theta }}_{j}^{w,(i)}$ as in (7). We then use the asymptotic $\chi ^2$-distribution to test the null hypothesis that the parameters of our model are equal to the parameters obtained from the England and Wales populations. The *p*-values $P^{w,(i)}_j = P^{w,(i)}_j(\lambda )$ are then calculated as in step 4 in the previous section, and the null hypothesis is rejected in any scenario *j* for which $P^{w,(i)}_j < 0.05$, that is, the significance level of the test is 0.05.

The power of the LRT for any fixed alternative *i*, relative population size *w* and fixed $\lambda$ is the proportion of the simulated *p*-values which are less than 0.05, that is, we count the number of scenarios for which the null hypothesis is rejected. More specifically, we define the random variables

19 $$\begin{aligned} R^{w,(i)}_j (\lambda )= & {} \left\{ \begin{array}{rl} 1 &{} \text{ if } P^{w,(i)}_j(\lambda ) < 0.05 \text{( }H_0 \text{ rejected) }\\ 0 &\text{ otherwise } \end{array} \right. \nonumber \\ R^{w,(i)}(\lambda )= & {} \frac{1}{N} \sum _{j=1}^N R^{w,(i)}_j (\lambda ) \end{aligned}$$

so that $R^{w,(i)}(\lambda )$ is the proportion of scenarios in which the null hypothesis is rejected among *N* simulated scenarios. We call $R^{w,(i)}(\lambda )$ the empirical rejection rate. Since we are considering independent scenarios, $R^{w,(i)}(\lambda )$ has a Binomial distribution,

20 $$\begin{aligned} N R^{w,(i)}(\lambda ) \sim \text {Bin}\Big (N, p^{w,(i)}(\lambda ) \Big ) \end{aligned}$$

where $p^{w,(i)}(\lambda )$ is the (unknown) power of the LRT if alternative $\theta ^{(i)}$ with parameter $\lambda$ is the true parameter set for the simulated death counts. Therefore, the empirical rejection rate $R^{w,(i)}(\lambda )$ is an unbiased estimator for the power $p^{w,(i)}(\lambda )$ and the estimated standard deviation of $R^{w,(i)}(\lambda )$ can easily be found from (20) in the usual way.

Then we investigate sensitivity of the power with respect to the size of $\lambda$ and the size of population *w*. for each of the four cases, $\theta ^{(1)},\ldots , \theta ^{(4)}$, we consider a set of values for $\lambda$ that are regularly spaced.

**Fig. 12:**
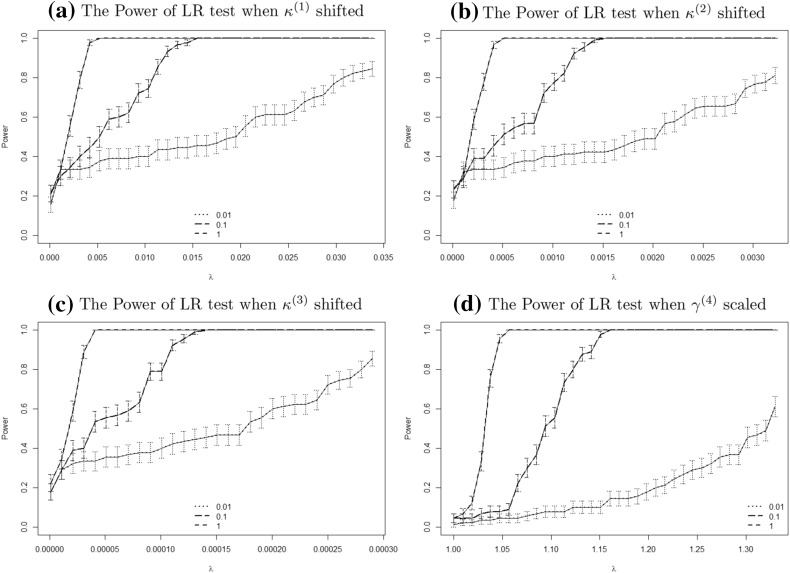
The empirical rejection rates $R^{w,(i)}(\lambda )$ under the LRT together with *error bars* for relative population sizes $w=1$ (*dashed line*), $w=0.1$ (*long dashed line*) and $w=0.01$ (*dotted line*). The width of the *error bars* is one standard error based on (20)

Figure 12 shows the obtained estimates $R^{w,(i)}_j(\lambda )$ for the power as a function of $\lambda$ for different relative population sizes *w*. Note that for each alternative $\theta ^{(i)}$ and any fixed $\lambda$ we have simulated $N=100$ scenarios, which is less than in the previous section. The reason is that we need to simulate those scenarios for each combination of *i* (alternative) and $\lambda$, which makes the total number of simulated scenarios very large.

Unsurprisingly, the power of the LRT is increasing in $\lambda$ for any $\theta ^{(i)}$ and relative population size *w*; the more we shift/scale the null hypothesis, the easier it is for the test to detect any shift/scaling. For the three period effects, decreasing the population size will greatly reduce the capability of LRT to detect the same amount of shift to a single parameter. We can also compare these plots with the earlier Fig. 2 which includes distributions of parameter estimates resulting from sampling variation. By way of example, for $w=0.01$ the width of the confidence interval in Fig. 2e for $\kappa ^{(3)}_{t,w}$ is about 0.005. This is much larger than the shifts that are considered in the power plot in Fig. 12. The reason why the latter values are so much lower is because we apply a systematic adjustment to all of the $\kappa ^{(3)}_{t,w}$, in contrast to random adjustments (due to sampling variation) in the former.

## Impact of parameter misspecification on mortality rates and annuities

We now investigate how significant the impact of shifting and scaling parameters is on the fitted mortality rates and corresponding annuity prices. We consider again the four alternatives in the previous section. For each of those and for each relative population size *w* we determine the value of $\lambda$ that results in a power of 50% of the LRT, that is, there is a 50% probability that the LRT will detect the wrong model and reject the null hypothesis. Those values are denoted by $\lambda ^{w,(i)}_{0.5}$ and shown in Table 3.

**Table 3:** The table contains the size of shift required for 50$\%$ power when each parameter is shifted separately, with respect to population $w=1,0.1,0.01$

Parameter shifted	$w=1$	$w=0.1$	$w=0.01$
$\lambda ^{w,(1)}_{0.5}$	0.003	0.006	0.02
$\lambda ^{w,(2)}_{0.5}$	0.0003	0.0006	0.002
$\lambda ^{w,(3)}_{0.5}$	0.0000025	0.000005	0.00018
$\lambda ^{w,(4)}_{0.5}$	1.03	1.09	1.32

We then calculate fitted mortality rates using the model in (2) and (3) with the following parameter constellations:

- $$\theta ^{w,(1)}_{0.5}=({\hat{\kappa }}_0^{(1)}+\lambda ^{w,(1)}_{0.5},{\hat{\kappa }}_0^{(2)},{\hat{\kappa }}_0^{(3)},{\hat{\gamma }}_0^{(4)})$$
- $$\theta ^{w,(2)}_{0.5}=({\hat{\kappa }}_0^{(1)}, {\hat{\kappa }}_0^{(2)}+\lambda ^{w,(2)}_{0.5},{\hat{\kappa }}_0^{(3)},{\hat{\gamma }}_0^{(4)})$$
- $$\theta ^{w,(3)}_{0.5}=({\hat{\kappa }}_0^{(1)}, {\hat{\kappa }}_0^{(2)},{\hat{\kappa }}_0^{(3)}+\lambda ^{w,(3)}_{0.5},{\hat{\gamma }}_0^{(4)})$$
- $$\theta ^{w,(4)}_{0.5}=({\hat{\kappa }}_0^{(1)}, {\hat{\kappa }}_0^{(2)},{\hat{\kappa }}_0^{(3)},\lambda ^{w,(4)}_{0.5}{\hat{\gamma }}_0^{(4)})$$

**Fig. 13:**
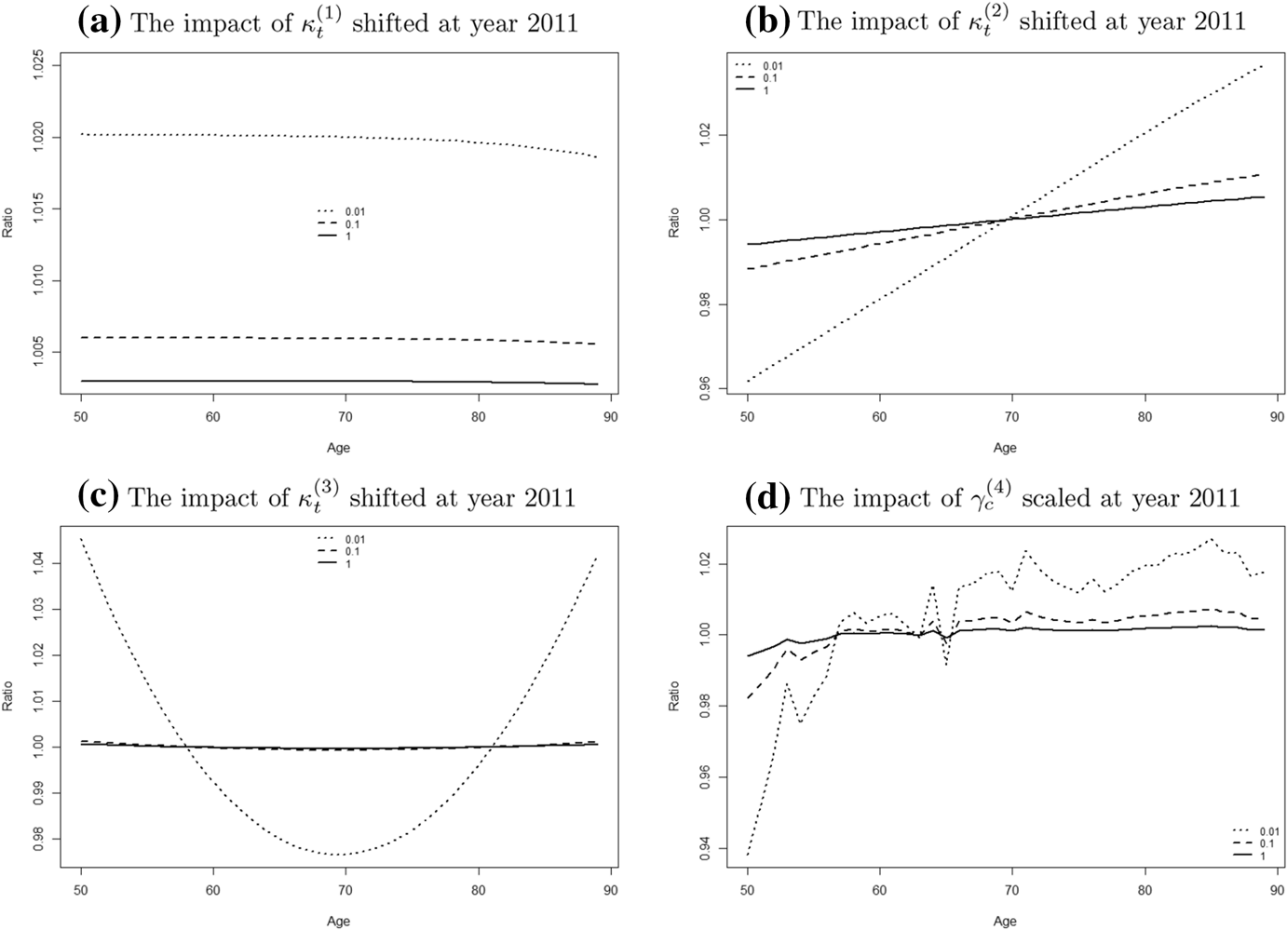
The impact of shifting each parameter separately on the estimated death rate of England and Wales. The shift is determined when it results in $50\%$ power for each population $w=1$ (*solid line*), $w=0.1$ (*dashed line*) and $w=0.01$ (*dotted line*)

To quantify the change in fitted mortality rates we calculate the following ratio

$$\begin{aligned} \rho _{t,x}^{w,(i)} = \frac{m(\theta ^{w,(1)}_{0.5},t,x)}{m({\hat{\theta }}_0,t,x)} \end{aligned}$$

for each $i = 1,2,3,4$ and different values of *w*. We expect that shifting $\kappa _t^{(1)}$, $\kappa _t^{(2)}$ and $\kappa _t^{(3)}$ will result in a parallel shift upwards, tilting rates in an anti clockwise direction and add some concavity to the rates respectively. This can indeed be seen in Figure 13 where we plot the ratio $\rho _{t,x}^{w,(i)}$ for the year $t=2011$. Figure 13d suggests that scaling $\gamma _c^{(4)}$ tilts and introduces more fluctuation to the ratio. For all the four parameters, reducing the relative population size *w* increased the relative change $\rho _{t,x}^{w,(i)}$ since $\lambda ^{w,(i)}_{0.5}$ increases. This confirms the intuitive idea that even misspecified parameters which produce significant changes in the fitted mortality rates are hard to detect with an LRT when the exposures are small.

From a financial point of view the effect on fitted mortality rates is only relevant in so far as annuity prices are affected. We will therefore consider the following annuities and discuss the effect of the four alternatives specified above on their values:

- A temporary annuity of £1 per annum payable annually in arrears to a life now aged 65 exactly, starting at the beginning of year 2012 with term of 25 years. Its expected present value is calculated as:
- An annuity of £1 per annum payable annually in arrears to a life now aged 55 exactly, deferred for 10 years, starting at the beginning of year 2012 with term of 25 years. Its expected present value is:

where *v* is the discount factor, $S(T+t,x)$ is the survival index for the probability of an individual aged *x* exactly at the start of year *T*, that will survive for the next *t* years. We assume the interest rate of $i=2\%$. The reason for investigating the deferred annuity is that Fig. 2g suggests that the estimates of cohort effect at $c=1946$ is approximately zero and the effect of scaling cohort estimates may not be obvious on the annuity price  but more obvious for .

We project the period and cohort effects in $\theta ^{w,(i)}_{0.5}$ ($i=1,2,3,4$) and ${\hat{\theta }}^{EW}$ forward for 35 years as in Sect. 4 where we use the point estimates defined in (9) and (10) for the parameters of the random walk for the shifted period effects, that is, we do not consider uncertainty about the drift and variance matrix of the random walk. Annuity prices are calculated for each sample path and we then calculate the average annuity price for each *w* with the *i*th parameter shifted or scaled. The results are shown in Tables 4 and 5.

The effects of shifting the period effects and scaling the cohort effect are somewhat varied. As might be expected, the impact on prices is most obvious for $w=0.01$. The impact on both types of annuity is straightforward to see for $\kappa ^{(1)}$: the shift pushes up mortality rates at all ages and lowers prices. For $\kappa ^{(2)}$ there is more impact on the age-65 annuity than the age-55 deferred annuity as the shift lowers mortality at younger ages and raises it at higher ages. For $\kappa ^{(3)}$, also, the impact is different at different ages. Finally, for $\gamma ^{(4)}$, the impact of scaling simply depends on the sign and magnitude of the value of $\gamma ^{(4)}$ for the cohort being priced.

Generally shifting or scaling the parameter estimates has no obvious effect on the annuity price and smaller populations can be affected more. Thus for testing a null hypothesis $H_0: \theta _r^{w=0.01}={\hat{\theta }}^{EW}$, if we accept $H_0$ when, in fact, they are actually different (type II error) the financial consequence of this type II error will be small in our case. In other words, the fact that we have accepted $H_0$ means that $\theta _r^{w=0.01}$, while not identical, must be very close to ${\hat{\theta }}^{EW}$, and that, therefore, any error in pricing will also be very small.

**Table 4:** The impact of shifting each parameter separately on the price of a twenty five-year temporary annuity for an individual aged at 65

Parameter shifted	England and Wales	$w=1$	$w=0.1$	$w=0.01$
$\kappa ^{(1)}$	14.67466	14.66393	14.65318	14.60280
$\kappa ^{(2)}$	14.67466	14.66887	14.66307	14.63588
$\kappa ^{(3)}$	14.67466	14.67500	14.67534	14.69850
$\gamma ^{(4)}$	14.67466	14.66997	14.66056	14.62441

The shift is determined when it results in $50\%$ power for each population $w=1, 0.1, 0.01$, which are shown in Table 3. We assume an interest rate of $2\%$

**Table 5:** The impact of shifting each parameter separately on the price of a ten-year deferred twenty five-year temporary annuity for an individual aged at 55

Parameter shifted	England and Wales	$w=1$	$w=0.1$	$w=0.01$
$\kappa ^{(1)}$	11.96545	11.95599	11.94652	11.84214
$\kappa ^{(2)}$	11.96545	11.96358	11.96169	11.95266
$\kappa ^{(3)}$	11.96545	11.96565	11.96584	11.97920
$\gamma ^{(4)}$	11.96545	11.96815	11.97355	11.99411

The shift is determined when it results in $50\%$ power for each population $w=1, 0.1, 0.01$, which are shown in Table 3. We assume an interest rate of $2\%$

## Likelihood ratio test for the cohort effect

The general form of the LRT as reviewed in Sect. 5.1 allows us to test a null hypothesis about parts of the parameter vector $\theta$ (restricted by the specified identifiability constraints as part of the model) rather than the entire $\theta = (\kappa _t^{(1)},\kappa _t^{(2)},\kappa _t^{(3)},\gamma _c^{(4)})$. Testing parts of $\theta$ is particularly relevant if mortality rates in a rather small population are modelled using estimated period or cohort effects from a larger population. Setting one or more of the components of $\theta$ equal to the function of corresponding parameters estimated from the large population reduces the dimension of the parameter vector which needs to be estimated from the small population where parameter uncertainty is rather strong as we have seen in Sect. 3. The example we have in mind is a pension fund that uses national mortality data to improve its mortality models, or when the mortality experience in a small country is modelled based on the combined experience of other similar countries.

In the reminder of this section we will use the LRT to test a null hypothesis about the cohort effect $\gamma$. In our general setting of Sect. 5.1 this means that

$$\begin{aligned} \theta _r = \gamma \text{ and } \theta _s = (\kappa _t^{(1)},\kappa _t^{(2)},\kappa _t^{(3)}) . \end{aligned}$$

Our null hypothesis is then that $\gamma = \gamma _0$ where $\gamma _0$ is a given vector of cohort effects, for which we later use an estimated cohort effect from a different population. We can now write the hypotheses as in (14) and proceed as in Sect. 5.2 to find the distribution of the LRT statistic in (17) for a finite sample of death counts from a small population.

For practical relevance we base our simulation study on the female and male populations in England and Wales. We choose $\gamma _0 = {\hat{\gamma }}^{EW}$, which is the estimated cohort effect from the mortality data for males in England and Wales. It is worth noting that, as ${\hat{\gamma }}^{EW}$ already satisfies the identifiability constraints, the null hypothesis $H_0: \gamma =\gamma _0$ has no testability problems under the given identifiability constraints defined in the model system. To investigate finite sample properties of $\Gamma$ we will need to specify a full parameter vector $\theta$ to simulate scenarios for the death counts. Having fixed the cohort effect $\gamma _0$ we choose the period effects to be the estimated period effects from data for the female population in England and Wales assuming that the cohort effect for those data is actually $\gamma _0$. As we are mainly interested in small populations we will consider deaths count scenarios for populations which have exposures equal to $wE_0$ where $E_0$ is here the exposure for the female population in England and Wales.

More specifically, we first find the MLE ${\tilde{\theta }}_s = \text{ arg } \text{ max }_{\theta _s} L(x~|~\theta _{r0} = \gamma _0,\theta _s)$ of the period effects $\theta _s = (\kappa _t^{(1)},\kappa _t^{(2)},\kappa _t^{(3)})$ from data for females assuming that the cohort effect is indeed $\gamma _0$ (which is the estimated cohort effect for males), see (16). Note that no constraints are applied for finding ${\tilde{\theta }}_s$ since the cohort is fixed and therefore there is no identifiability problem. We then generate *N* realisations of the value of the test statistic $\Gamma ^w$ for different values of the relative population size *w* using the following algorithm:

1. Simulate death counts $D^w_j$ as in (6) using the parameter vector
  $$\begin{aligned} {\tilde{\theta }}= ({\tilde{\theta }}_s, \theta _{r0}) = ({\tilde{\kappa }}_t^{(1)},{\tilde{\kappa }}_t^{(2)},{\tilde{\kappa }}_t^{(3)}, \gamma _0) \end{aligned}$$
  to obtain scenarios $D^w_j$ for different values of the relative population size *w*. The period effects ${\tilde{\kappa }}$ are estimated from data for females with the cohort effect fixed to $\gamma _0$. The exposure is $wE_0$ where $E_0$ is the exposure for the female population in England and Wales.
2. Find the MLE ${\tilde{\theta }}_{s,j}$ of period effects $\kappa$ in scenario *j* assuming that the null hypothesis holds, as in (16).
3. Find the unrestricted MLE ${\hat{\theta }}_j$ as in (15).
4. Calculate the value of the test statistic $\Gamma ^w_j$ in (17) in each scenario *j*.
5. Calculate the *p*-values $P^w_j$ based on the asymptotic $\chi ^2$-distribution with $\alpha$ degrees of freedom, where $\alpha$ is the number of parameters (cohorts) *r* minus the number of constraints as in Sect. 5.2. For our data set we obtain $\alpha = 87$.

**Fig. 14:**
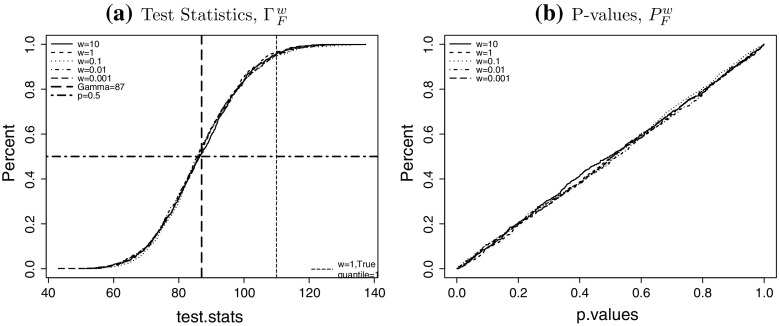
The results of likelihood ratio test, with distributions of test statistics (**a**) and p-values (**b**), for the population of $w=10$ (*solid line*), $w=1$ (*dashed line*), $w=0.1$ (*dotted line*), $w=0.01$ (*dashed dotted line*) and $w=0.001$. The *left vertical dashed line* is the mean of normal approximation for the $\chi _{87}^2$, at $x=87$. The *right dashed line* at $x=110$ is the true $95\%$ quantile of population $w=1$

The simulated distribution functions of the LRT statistic $\Gamma ^w$ and the *p*-values $P^w$ are shown in Fig. 14. The results suggest that changing the size of the population has no significant impact on the distribution of $\Gamma ^w$ and that the *p*-values are roughly uniformly distributed for all *w*, which is an indication that the $\chi ^2$-approximation works well for our data set as we have also found in Sect. 5.2 where the full parameter vector was tested.

## Empirical examples

We apply the LRT for the cohort effect in two empirical studies.

### Females vs. males in England and Wales

The population for which we wish to test the cohort effect first is the female population in England and Wales that we already considered in our simulation study. Our null hypothesis is therefore that the true cohort effect for the female population in England and Wales is equal to the estimated cohort effect for males in England and Wales. Note that this is different from testing the hypothesis that the male and female population share the same (true) cohort effect since we ignore the uncertainty about the estimated cohort effect for males.

**Fig. 15:**
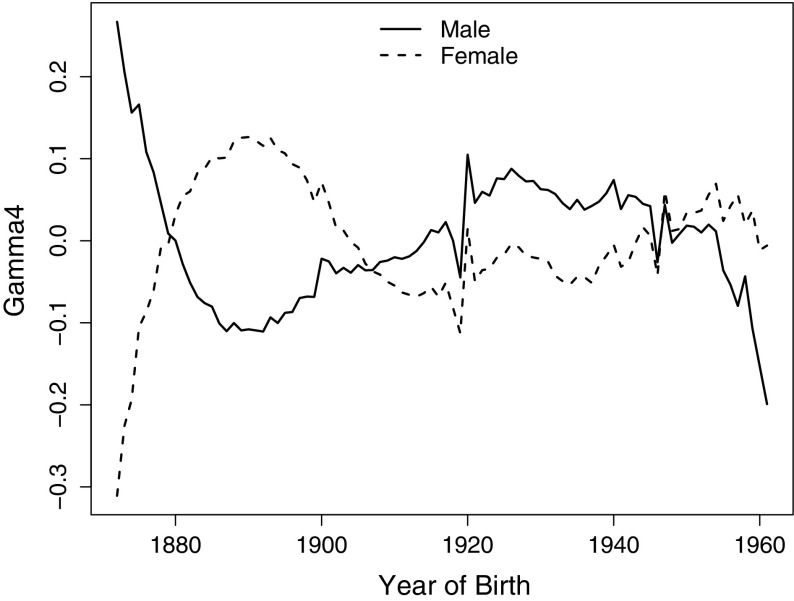
The estimates of cohort effect, for England and Wales males (*solid line*) and females (*dashed line*), age 50 to 89 last birthday, over year 1961 to 2011

To illustrate the difference between the two cohort effects we plot in Fig. 15 the estimated cohort effects for females and males. There are fairly strong similarities between the two curves after about 1910, but there are also significant qualitative differences before 1900. To check empirically, that these differences are not simply the result of the identifiability constraints, one can plot ${\hat{\gamma }}^{(4),M}-{\hat{\gamma }}^{(4),F}$. If this looks quadratic then the differences could, simply, be due to the identifiability constraint. But for these data, a plot of ${\hat{\gamma }}^{(4),M}-{\hat{\gamma }}^{(4),F}$ would clearly not be quadratic (exhibiting more of a cubic shape).

This difference can be confirmed more formally using the LRT with the null hypothesis that the females have the same cohort effect as the previously estimated males cohort effect. The test statistic $\Gamma$ is approximately 6311, which is an extremely high value for a $\chi ^2$-distribution with 87 degrees of freedom and is also very high compared to the values of $\Gamma$ observed in our simulation study, see Fig. 14. The *p*-value is therefore very close to zero, and we reject the null hypothesis that the cohort effect fro the mortality of the female population is the same as the previously estimated cohort effect for the male population.

### Male mortality in Scotland vs. England and Wales

**Fig. 16:**
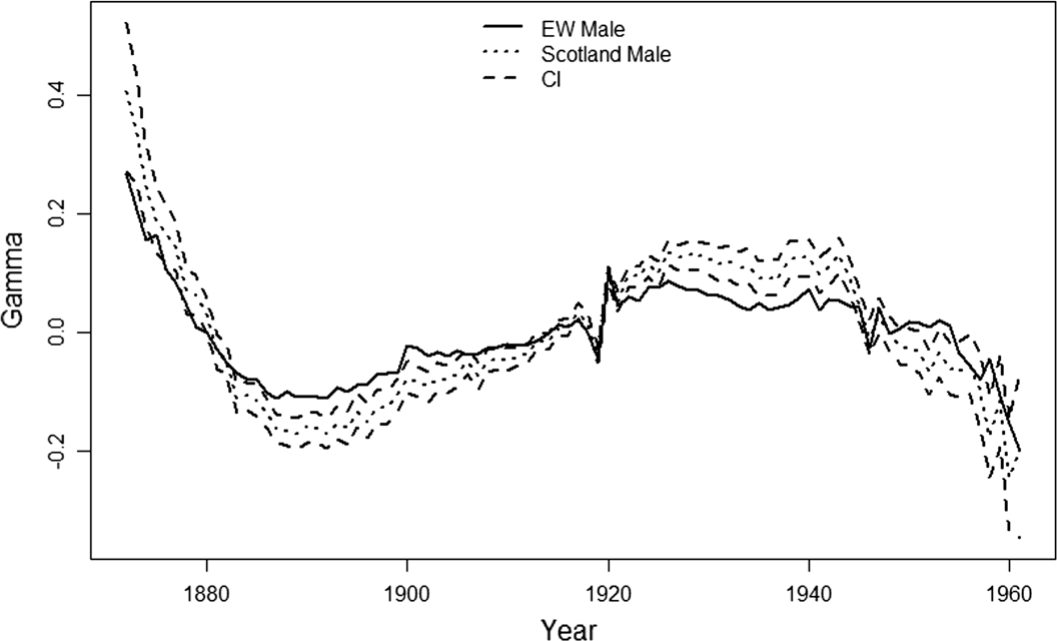
The estimates of cohort effect, for the males of England and Wales (*solid line* ) and Scotland (*dotted line*), age 50 to 89 last birthday, over year 1961 to 2011. The dashed lines are the CI for the cohort effect of Scotland. The upper bound is 95% quantile of the distribution and the lower bound is 5% quantile

A second, and more intriguing, empirical example concerns the cohort effects estimated from mortality data for the male population in England and Wales versus the male population in Scotland. Figure 16 compares the independently-estimated cohort effects with a confidence interval added around the Scottish estimates. Compared to Fig. 15, the two curves here look much more similar, with the pattern of ${\hat{\gamma }}^{(4),EW}-{\hat{\gamma }}^{(4),S}$ again not like a quadratic function with respect to cohort year *c*. On the other hand, we find that most of the cohort effects for males in England and Wales lie outside of the

confidence interval calculated for Scottish males. This suggests that although the two populations have similar pattern for the cohort estimates, the difference might still be significant.

For the LRT we again choose $\gamma _0= {\hat{\gamma }}^{EW}$ and then test the hypothesis that the true cohort effect for Scottish males is equal to $\gamma _0$. The 99% quantile of a $\chi ^2$-distribution with 87 degrees of freedom is approximately 121. For the test statistic we find $\Gamma = 193.37$ and we therefore reject the null hypothesis and conclude that the cohort effect in Scotland is significantly different from the estimated cohort effect for England and Wales. This indicates that there might be factors in the Scottish male population that result in significant differences throughout time. However, we might speculate that there is a common cohort effect, that is, for some reason, magnified in Scotland. Investigating this in detail is beyond the scope of this paper, but we speculate that a magnified effect might be the result of socio-economic differences between the two populations: for example, cohort effects might be greater in lower socio-economic groups.

## Conclusion

In this paper, we investigated the finite sample distribution of the maximum likelihood estimators for the parameters of a stochastic mortality model. We found that the size of a population has a significant effect on the uncertainty about the estimated parameters and mortality projections. In particular, we found that there exists a bias in the estimated covariance matrix of the random walk fitted to the period effects when the size of the underlying population is small. As a consequence, prediction intervals are rather wide for small populations even when parameter uncertainty is ignored.

To investigate if parameters estimated from larger populations can be used to generate scenarios for smaller populations we investigated how a likelihood ratio test performs when applied to the mortality experience of a small population. We found that the finite sample distribution of the test statistic is very close to the asymptotically correct $\chi ^2$ distribution and, therefore, the observed rejection rates are close to the chosen significance level. However, we also found that the power of the test depends strongly on the population size with the ability of the test to detect deviations from the null hypothesis being significantly reduced when the size of the underlying populations is small.

A brief investigation of annuity prices has shown that the misspecification of parameters has a limited financial impact. Considering shifts in the parameter values which the LR test would detect with a $50\%$ chance we have seen that the impact of a small population size is significant for deferred annuities. To have a complete picture of possible further financial consequences, a more detailed study is required, which is beyond the scope of this paper.

In our empirical analysis we then applied the LRT, and found that neither of the mortality rates of the female population in England and Wales and the male population in Scotland should be modelled with a cohort effect estimated from the male population in England and Wales.

In this paper, we used the traditional two-stage fitting approach whereby the period and cohort effects are estimated using the Poisson maximum likelihood method in the first stage and a time series model is fitted to these effects in the second stage. We have found that sampling variation in the small population datasets has significant impact, which can then obscure the true signal in those effects, and giving rise to misleading forecasts. Bayesian approaches that combines the two stages into one, e.g., [29], Cairns et al. (2011) and [12]) can be used to provide a way to address this problem. However, as use of the two-stage approach is widespread (perhaps because of its relative simplicity) we have, here, attempted the first systematic analysis of the impact of population size on parameter estimates and forecasts using the two-stage approach. In this way, users of the two-stage approach will be better informed about its limitations as well as understanding how the likelihood ratio test might be used to exploit data from larger populations.

## Derivation of the asymptotic distribution of $\theta _w$

Recall the log likelihood function

$$\begin{aligned} l(\theta _w,D_{t,x}^{w},E_{t,x}^{w})=\sum _{t,x}f(t,x,\theta _w)-g(t,x,\theta _w)+h(t,x) \end{aligned}$$

where

$$\begin{aligned} f(t,x,\theta _w)= & {} D_{t,x}^w\text {log}[m(t,x,\theta _w)]\\ g(t,x,\theta _w)= & {} wE_{t,x}^wm(t,x,\theta _w) \end{aligned}$$

and *h*(*t*, *x*) is not a function with respect to $\theta _w$. The form of the column vector $\theta _w$ with $4ny+na-1$ dimensions is

$$\begin{aligned} \theta _w=(\kappa _{t_1,w}^{(1)},\ldots ,\kappa _{t_{ny},w}^{(1)},\kappa _{t_1,w}^{(2)},\ldots ,\kappa _{t_{ny},w}^{(2)},\kappa _{t_1,w}^{(3)},\ldots ,\kappa _{t_{ny},w}^{(3)},\gamma _{c_1,w}^{(4)},\ldots ,\gamma _{c_{ny+na-1},w}^{(4)})^T \end{aligned}$$

The second derivative of $l(\theta _w,D_{t,x}^{w},E_{t,x}^{w})$ is

$$\begin{aligned} \frac{\partial ^2 l}{\partial \theta _w^2}=\sum _{t,x}\frac{\partial ^2 f}{\partial \theta _w^2}-\frac{\partial ^2 g}{\partial \theta _w^2} \end{aligned}$$

For every pair of (*t*, *x*), $m(t,x,\theta _w)$ is a single value, thus the second derivative of *f* and *g* with respect to $\theta _w$ is a Hessian matrix with $4ny+na-1$ rows and columns, with the form as

$$\begin{aligned} \frac{\partial ^2 f}{\partial \theta _w^2}= & {} \left( \begin{array}{ccccc} \frac{\partial ^2 f}{\partial \kappa _{t_1,w}^{(1)~2}}&{}\dots &{}\frac{\partial ^2 f}{\partial \kappa _{t_1,w}^{(1)} \partial \kappa _{t_n,w}^{(i)}}&{}\dots &{}\frac{\partial ^2 f}{\partial \kappa _{t_1,w}^{(1)} \partial \gamma _{c_{ny+na-1},w}^{(4)}}\\ \vdots &{}\vdots &{}\vdots &{}\vdots &{}\vdots \\ \vdots &{}\vdots &{}\vdots &{}\vdots &{}\vdots \\ \frac{\partial ^2 f}{\partial \gamma _{c_{ny+na-1},w}^{(4)}\partial \kappa _{t_1,w}^{(1)}}&{}\dots &{}\frac{\partial ^2 f}{\partial \gamma _{c_{ny+na-1},w}^{(4)} \partial \kappa _{t_n,w}^{(i)}}&{}\dots &{}\frac{\partial ^2 f}{ \partial \gamma _{c_{ny+na-1},w}^{(4)^2}}\\ \end{array}\right) \end{aligned}$$

Thus the form of the element at row *m* and column *n* is

$$\begin{aligned} \frac{\partial ^2 f}{\partial \kappa _{m,w}^{(i)}\partial \kappa _{n,w}^{(j)}}=-\frac{D_{t,x}^w}{m(t,x,\theta _w)^2}\frac{\partial m(t,x,\theta _w)}{\partial \kappa _{m,w}^{(i)}}\frac{\partial m(t,x,\theta _w)}{\partial \kappa _{n,w}^{(j)}}+\frac{D_{t,x}^w}{m(t,x,\theta _w)}\frac{\partial ^2 m(t,x,\theta _w)}{\partial \kappa _{m,w}^{(i)}\partial \kappa _{n,w}^{(j)}} \end{aligned}$$

where $i,j\in (1,2,3,4)$ and are not necessarily the same. Note that $\kappa ^{(4)}$ represents the cohort effect for convenience. Same derivation can be done for *g*, and we have

$$\begin{aligned} \frac{\partial ^2 f}{\partial \kappa _{m,w}^{(i)}\partial \kappa _{n,w}^{(j)}}=wE_{t,x}^w\frac{\partial ^2 m(t,x,\theta _w)}{\partial \kappa _{m,w}^{(i)}\partial \kappa _{n,w}^{(j)}} \end{aligned}$$

Thus for each pair of (*t*, *x*), the expected value of the element of the second derivative of *l* is

$$\begin{aligned} E\left[ \frac{\partial ^2 (f-g)}{\partial \kappa _{m,w}^{(i)}\partial \kappa _{n,w}^{(j)}}\right] =-w\frac{E_{t,x}^w}{m(t,x,\theta _w)}\frac{\partial m(t,x,\theta _w)}{\partial \kappa _{m,w}^{(i)}}\frac{\partial m(t,x,\theta _w)}{\partial \kappa _{n,w}^{(j)}} \end{aligned}$$

Thus we have the fisher information matrix given ${\hat{\theta }}^{EW}$ as

$$\begin{aligned} I({\hat{\theta }}^{EW})=w\sum _{t,x}\frac{E_{t,x}^{EW}}{m(t,x,{\hat{\theta }}^{EW})}\left[ \frac{\partial m(t,x,\theta _w)}{\partial \theta _w}\left( \frac{\partial m(t,x,\theta _w)}{\partial \theta _w}\right) ^T\right] \Bigg |_{\theta _w={\hat{\theta} ^{EW}}}. \end{aligned}$$

Further, given $$t=t_m, x=x_n$$,

$$\begin{aligned} \frac{\partial m(t_m,x,\theta _w)}{\partial \theta _w}=(\frac{\partial m(t_m,x_n,\theta _w)}{\partial q(\theta _w,t_m,x_n)}\frac{\partial q(\theta _w,t_m,x_n)}{\kappa _{t,w}^{(1)}}{\mathbbm{1}}_{t=t_m}\dots \frac{\partial m(\theta _w,t_m,x_n)}{\partial q(\theta _w,t_m,x_n)}\frac{\partial q(\theta _w,t_m,x_n)}{\kappa _{t,w}^{(4)}}{\mathbbm{1}}_{t=t_m}) \end{aligned}$$

where

$$\begin{aligned} {\mathbbm{1}}_{t=t_m}=\Big \{ \begin{array}{cc} 1&{} t=t_m\\ 0&{} \text {otherwise.} \end{array} \end{aligned}$$

## Footnotes

1 Human Mortality Database. University of California, Berkeley (USA), and Max Planck Institute for Demographic Research (Germany). Available at *www.mortality.org *or *www.humanmortality.de* (data downloaded on 16 February, 2014).

2 See [19] for more details about the likelihood ratio test and the asymptotic distribution of the LRT statistic.

3 See [32] for more details about testable hypotheses.

4 See [15] for more details on statistical power.
